# Local and foreign authorship of maternal health interventional research in low- and middle-income countries: systematic mapping of publications 2000–2012

**DOI:** 10.1186/s12992-016-0172-x

**Published:** 2016-06-23

**Authors:** Matthew F. Chersich, Duane Blaauw, Mari Dumbaugh, Loveday Penn-Kekana, Ashar Dhana, Siphiwe Thwala, Leon Bijlmakers, Emily Vargas, Elinor Kern, Francisco Becerra-Posada, Josephine Kavanagh, Priya Mannava, Langelihle Mlotshwa, Victor Becerril-Montekio, Katharine Footman, Helen Rees

**Affiliations:** Wits Reproductive Health and HIV Institute, University of the Witwatersrand, Johannesburg, South Africa; Centre for Health Policy/MRC Health Policy Research Group, School of Public Health, Faculty of Health Sciences, University of the Witwatersrand, Johannesburg, South Africa; International Centre for Reproductive Health, Department of Obstetrics and Gynecology, Ghent University, Ghent, Belgium; Independent Consultant, Department of Maternal, Newborn, Child and Adolescent Health, World Health Organization, Geneva, Switzerland; Swiss Tropical and Public Health Institute, Department of Epidemiology and Public Health, Society, Gender and Health Unit, Basel, Switzerland; Department of Infectious Disease Epidemiology, London School of Hygiene and Tropical Medicine, London, UK; Radboud University Medical Center, Radboud Institute for Health Sciences (RIHS), Nijmegen, The Netherlands; Innovation in Public Health Department, National Institute of Health, Bogotá D.C., Colombia; Centre for Health Systems Research/National Institute of Public Health (Instituto Nacional de Salud Pública), Cuernavaca, México; Pan American Health Organization, Washington, D.C., USA; Centre for International Health, Burnet Institute, Melbourne, VIC Australia; London School of Hygiene & Tropical Medicine, London, UK

**Keywords:** LMICs, Authorship, Maternal health, Systematic review, Research governance

## Abstract

**Background:**

Researchers in low- and middle-income countries (LMICs) are under-represented in scientific literature. Mapping of authorship of articles can provide an assessment of data ownership and research capacity in LMICs over time and identify variations between different settings.

**Methods:**

Systematic mapping of maternal health interventional research in LMICs from 2000 to 2012, comparing country of study and of affiliation of first authors. Studies on health systems or promotion; community-based activities; and haemorrhage, hypertension, HIV/STIs and malaria were included. Following review of 35,078 titles and abstracts, 2292 full-text publications were included. Data ownership was measured by the proportion of articles with an LMIC lead author (author affiliated with an LMIC institution).

**Results:**

The total number of papers led by an LMIC author rose from 45.0/year in 2000–2003 to 98.0/year in 2004–2007, but increased only slightly thereafter to 113.1/year in 2008–2012. In the same periods, the proportion of papers led by a local author was 58.4 %, 60.8 % and 60.1 %, respectively. Data ownership varies markedly between countries. A quarter of countries led more than 75 % of their research; while in 10 countries, under 25 % of publications had a local first author. Researchers at LMIC institutions led 56.6 % (1297) of all papers, but only 26.8 % of systematic reviews (65/243), 29.9 % of modelling studies (44/147), and 33.2 % of articles in journals with an Impact Factor ≥5 (61/184). Sub-Saharan Africa authors led 54.2 % (538/993) of studies in the region, while 73.4 % did in Latin America and the Caribbean (223/304). Authors affiliated with United States (561) and United Kingdom (207) institutions together account for a third of publications. Around two thirds of USAID and European Union funded studies had high-income country leads, twice as many as that of Wellcome Trust and Rockefeller Foundation.

**Conclusions:**

There are marked gaps in data ownership and these have not diminished over time. Increased locally-led publications, however, does suggest a growing capacity in LMIC institutions to analyse and articulate research findings. Differences in author attribution between funders might signal important variations in funders’ expectations of authorship and discrepancies in how funders understand collaboration. More stringent authorship oversight and reconsideration of authorship guidelines could facilitate growth in LMIC leadership. Left unaddressed, deficiencies in research ownership will continue to hinder alignment between the research undertaken and knowledge needs of LMICs.

**Electronic supplementary material:**

The online version of this article (doi:10.1186/s12992-016-0172-x) contains supplementary material, which is available to authorized users.

## Background

Researchers in low- and middle-income countries (LMICs) are under-represented in scientific literature as a whole, and in fora such as editorial boards of journals [1–7]. Even within fields particularly pertinent to LMICs, such as tropical medicine, only around five percent of articles are written exclusively by authors from countries with a low human development index, and an equally low percentage of editorial and advisory boards for journals in this field include people from these countries [1]. The proportion of studies in LMICs where the lead author is affiliated with an institution in a high-income country (HIC) does, however, vary considerably between research fields. For example, HIC authors led 50–52 % of studies done on international epidemiology, psychiatry and tropical medicine in LMICs [1, 5, 8], while they led only about 10 % of publications on orthopaedics [9] or on tuberculosis and lung disease in LMICs [10]. Reviews of publishing patterns at country level also demonstrate deficiencies in representation of LMIC authors. In Fiji, for example, in the period from 1965 to 2002, only 13.5 % of publications on health in the country had a Fijian first author [11]. By contrast, under one percent of studies in HICs included an LMIC author, and a similar proportion of scientists affiliated to an institution in one LMIC led articles reporting the findings of research done in another LMIC [8, 9].

For an individual, ownership of study results – based on active participation and research leadership – provides a powerful incentive for securing local dissemination and application of those results [12, 13]. Authorship also boosts one’s career prospects, motivation and opportunities for research funding. For institutions and countries, their capacity to lead research might reflect their ability to drive a local research agenda, tailored to the priorities of the country [12, 14]. These priorities might well differ from the interests of foreign researchers or external donors [15].

This study maps authorship of research outputs on maternal health in LMICs, and temporal shifts in authorship between LMICs and HICs. By examining the proportion of articles where the lead author is affiliated with an institution in the same country as the study, together with the characteristics of the articles, we aim to identify factors associated with data ownership, and the capacity to analyse and articulate research findings in LMICs. Based on the patterns identified, we also make inferences about the extent to which the considerable investment in North–south collaborations in recent decades has impacted on scientific leadership in LMICs. The data presented were extracted from full text articles included in a large systematic mapping of literature on maternal health interventions in LMICs. Mapping allows for a more comprehensive assessment than is done in classic bibliometric studies, which only summarise the data fields contained within databases such as Medline. The field of interventional research in maternal health was investigated given the continued high levels of maternal mortality in LMICs and the funding constraints for research on this topic [16, 17].

## Methods

### Identification of literature and database management

The review draws on a database of maternal health literature developed by the MASCOT/MHSAR project, which is a large-scale systematic mapping of all maternal health interventions in LMICs published between 01/01/2000 and 31/08/2012 [18]. A systematic mapping of a body of literature differs from a classic systematic review, which addresses a single, clearly-defined research question [19]. Systematic mapping identifies and describes all papers published on a broad topic, but does not assess the quality of the included research.

Identifying research publications in LMICs poses several challenges, as much of this research is published in journals which are not indexed by the major biomedical databases [20]. The search thus extended to regional databases and registers of research specific to LMICs, alongside the major biomedical databases. A sensitive search strategy was developed, combining controlled vocabulary and free-text terms. Search terms were finalised following several exploratory searches and piloting. Terms for maternal health were combined, where appropriate, with terms for LMICs. No language restrictions were employed. The initial search was conducted in MEDLINE and then adapted for: African Journals Online, African Index Medicus, Cumulative Index to Nursing and Allied Health Literature, Embase, Index Medicus for the South East Asia Region, LILACS, PopLINE, PsycINFO and Web of Knowledge (Science Citation Index Expanded, Social Sciences Citation Index). See Additional file 1.

To be included in the mapping, studies on maternal health in an LMIC had to address health system or promotion interventions, community-based interventions, or clinical interventions in LMICs on one of four tracer conditions: haemorrhage, hypertension, HIV and other sexually transmitted infections (STIs), or malaria. The four tracer conditions were selected as two of them constitute the most common causes of direct maternal deaths (haemorrhage and hypertension), while HIV and malaria are the principal causes of indirect maternal deaths in many LMICs [16]. Included were studies that targeted a maternal health population (women in pregnancy, childbirth or within two years postpartum), or male and female community members involved in a maternal health intervention. General health system interventions were included only if they reported outcomes in a maternal health population. We excluded articles related to fertility or infertility, and descriptions of coverage of routine services, given the difficulties in standardising data extraction from these studies across a large review team (14 reviewers across 6 countries). All study designs were eligible, aside from descriptive studies, narrative reviews and policy discussion papers. Studies could be in Arabic, English, French, Portuguese and Spanish.

**Fig. 1:**
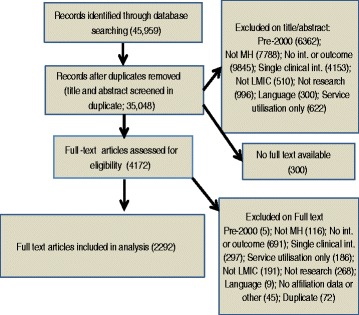
Flow diagram of overall systematic mapping

Management of the database, screening for eligibility and data extraction were done using online systematic review software (EPPI-Reviewer 4; http://eppi.ioe.ac.uk/). Of the 45 959 papers initially uploaded, 10 881 were duplicate items (Fig. 1). The titles and abstract of the remaining records (35 078) were screened independently by two reviewers. Differences between reviewers were resolved by a third reviewer. Of 4175 full text papers reviewed, 2292 were included in the final mapping. From the full text articles, we extracted data about the country(ies) where the study was done and the country(ies) of affiliation of the first author. Other variables extracted included study design, intervention topic and research funder. Data on the journal’s Impact Factor, used as a measure of quality of the publication, were downloaded from Thomson Reuters [21].

### Study variables and analysis

An article was classified as having a local lead author when the first author of the paper was affiliated with an institution based in the same country in which the study was done. Similarly, an author affiliated with an institution in a HIC was classified as an HIC author. Authors with multiple affiliations were categorised as being locally affiliated if one of the affiliations they held was in the country of study. For multi-country studies, we classified the publication as locally led if the first author held an affiliation in any of the study countries. If a study group was given as the first author, then the first person listed in the group was considered the lead. The proportion of articles with a local lead author was used as a measure of *data ownership*, which is an important component of overall ownership of research and of scientific leadership in a country. In turn, the total number of articles led by a local author serves as a proxy of the *capacity of researchers* to analyse and articulate research findings [14]. The list of LMICs and income sub-regions was based on the World Bank classification [22].

Individual funders were categorised into one of ten funder types, using a classification adapted from previous studies tracking official development assistance [17, 23]. To identify changes over time, annual rates of publications (n papers/year) were calculated for three time periods (2000–2003, 2004–2007 and 2008–2012). The last period consisted of 4.67 years, while the other two were each four years.

Data checks were performed in the online software and in Stata 13 (StataCorp LP, College Station, TX, USA); the latter also used for data analysis. Variations in lead author were examined between countries and regions, intervention topics, study designs and research funders. For systematic reviews and modelling studies, we assessed only the proportion of articles led by an LMIC author, and did not compare that to the country of research. This was done as studies with these designs mostly entail secondary analysis of existing publications, rather than the collection of empirical data within a country (the focus of the study presented here).

Chi square tests were used to detect associations between categorical variables. For continuous variables, an unpaired Student’s t test or Mann–Whitney U test compared data with a normal and non-normal distribution respectively. The study has multiple exposures and outcomes, which raises the likelihood of a Type 1 error (false ‘significance’). Adjusting the *P* value to take account of this would, however, increase the risk for Type 2 errors (the chance that true associations are not detected). As the study is descriptive and exploratory, we considered the risk of Type 2 errors to outweigh Type 1 [24]. However, for analysis comparing authorship between different countries, those countries with fewer than five publications were grouped together to avert random error incurred by very small samples. As multiple responses for country of author and of study were possible, totals may exceed the number of articles in the review, and the sum of percentages may exceed 100.

## Results

The analysis assessed authorship of 2292 articles, of which 49.1 % (1126) were led by an author affiliated at an LMIC institution, 43.4 % (995) by an HIC affiliate, and the remaining 7.5 % were authors who held affiliations at both an LMIC and HIC institution (171). The total number of papers led by an author affiliated with an LMIC institution (a measure of LMIC research capacity) rose from 45.0/yr in 2000–2003 to 98.0/yr in 2004–2007, but increased only slightly thereafter to 113.1/yr in 2008–2012. Comparing the first and last time periods, increments in numbers of locally-authored articles per year were particularly marked in the fields of HIV (15.0 to 42.8) and health systems (12.0 to 40.5), and in studies using qualitative methods (1.0 to 8.4) or systematic review (1.5 to 9.4, Table 1).

**Table 1:** Changes over time in lead authorship, by topics, study design and region

Variable (n)	2000–2003	2004–2007	2008–2012	*P* ^b^
	% local lead (n/year)	% local lead (n/year)	% local lead (n/year)	
TOTAL % (n)	58.4 (45)	60.8 (98)	60.1 (113.1)	0.74
Research topics				
Haemorrhage	55.2 (4)	54.6 (7.5)	56.9 (8.8)	0.83
Hypertension	90.3 (7)	76.7 (11.5)	86.0 (10.5)	0.86
HIV	59.4 (15)	56.0 (35.3)	61.7 (42.8)	0.39
STIs other than HIV	68.0 (4.3)	61.5 (6)	71.4 (4.3)	0.77
Malaria	82.4 (7)	69.2 (13.5)	61.7 (15.8)	0.03
Health Systems	42.1 (12)	52.9 (27)	52.8 (40.5)	0.09
EmOC	35.7 (2.5)	42.1 (6)	46.4 (8.4)	0.32
Demand-side Financing	75.0 (0.8)	58.3 (1.8)	51.2 (4.7)	0.35
Equity examined	54.6 (3)	50.9 (7.3)	62.6 (14.3)	0.23
TBA	13.3 (0.5)	65.2 (3.8)	42.2 (4.1)	0.25
Study design				
Effectiveness	60.0 (34.5)	63.8 (77.5)	60.2 (83.9)	0.72
Randomised Controlled Trials	56.1 (9.3)	56.0 (14)	63.9 (16.3)	0.25
Qualitative	66.7 (1)	56.1 (5.8)	58.2 (8.4)	0.92
Mixed Methods	16.7 (0.3)	16.7 (0.8)	50.0 (4.5)	0.01
Systematic Reviews^a^	22.2 (1.5)	23.4 (3.8)	29.1 (9.4)	0.32
Modelling Studies^a^	33.3 (2.5)	21.6 (2.8)	35.4 (4.9)	0.57
Multi-country study				
Multi-country study	42.1 (2)	43.5 (5)	41.0 (6.9)	0.86
Economic zone				
UMIC	81.8 (22.5)	78.5 (47.5)	80.5 (46.0)	0.94
LMIC	48.2 (10)	53.2 (25.3)	59.0 (36.4)	0.06
LIC	47.3 (13)	49.5 (26.5)	44.5 (32.3)	0.42
Geographical region				
East Asia Pacific	70.8 (8.5)	68.3 (10.8)	60.8 (12.6)	0.20
Europe, Central Asia	66.7 (1.5)	83.3 (5)	63.0 (3.6)	0.45
Latin America, Caribbean	70.2 (8.3)	70.6 (21)	76.4 (20.1)	0.31
Middle East, North Africa	16.7 (0.25)	50.0 (2.3)	70.4 (4.1)	0.01
South Asia	72.7 (6)	67.5 (13)	61.4 (17.3)	0.18
Sub-Saharan Africa	52.9 (20.8)	54.2 (46.8)	55.4 (55.9)	0.56
Journal with an Impact Factor				
Papers in journal with IF	49.6 (28)	49.5 (57.3)	49.5 (65.1)	1.0
Journal with IF 0–1.9	65.5 (9)	73.2 (20.5)	67.2 (27.6)	0.85
Journal with IF 2–4.9	28.9 (3.8)	42.4 (13.3)	41.3 (18.2)	0.22
Journal with IF ≥5	38.7 (3)	30.0 (4.5)	35.4 (6.2)	0.93

^a^Proportion of papers with an LMIC first author is presented for these study designs. ^b^Chi-square test for trend. The total number of papers in the last time period (01/2008–08/2012) was divided by 4.67 years, while the first 2 periods were each 4 years. *IF* Impact Factor (article in a journal that has an Impact Factor)

### Publications of empirical data by economic and geographical regions

**Fig. 2:**
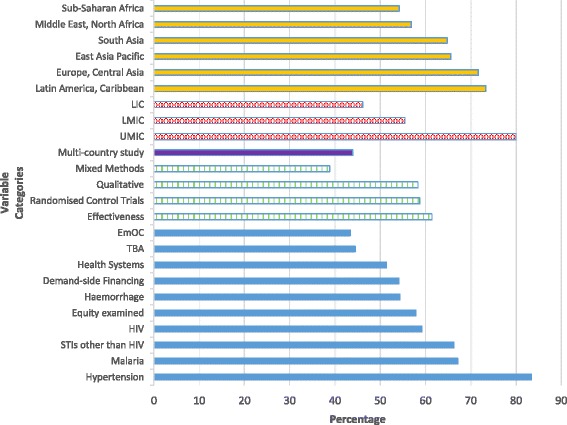
Percentage of articles with a local first author by region of the world, study design and topic

Of studies which were based on empirical data collected in an LMIC (excluding 416 systematic review or modelling studies), 60.0 % were locally led (1125/1876). This figure varied from about 80 % in upper middle-income countries, to 55.5 % in lower middle-income countries and 46.1 % in low-income countries (Fig. 2). The proportion of local authorship only increased over time in lower middle-income countries (48.9 % in 2000–2003 to 59.0 % in 2008–2012), but not in other economic zones. The proportion of publications with a local lead rose from 16.1 to 70.4 % in the Middle East, North Africa region. Sub-Saharan Africa had the lowest overall proportion of locally-led publications of all geographical regions (54.2 %; Table 2). These levels were considerably higher in Latin America and the Caribbean (73.4 %), and in Europe and Central Asia (71.7 %). Little evidence of cooperation between LMICs was identified; below one percent of studies in LMICs were led by a person holding an affiliation in an LMIC other than the country of study.

**Table 2:** Patterns of lead authorships by region

Regions (total n papers in region)	Local lead author % (n local)	Lead in papers in IF journal % (n local)	Lead in papers in journal with IF ≥2 % (n local)	Lead from another country in region (%)	North America lead author (%)	Europe lead author (%)	Australasia lead author (%)
East Asia Pacific (212)	65.6 (139)	68.8 (66)	69.5 (41)	0	18.4	19.8	8.0
Europe, Central Asia (60)	71.7 (43)	68.8 (22)	56.3 (9)	0	13.3	15.0	0
Latin America, Caribbean (304)	73.4 (223)	72.2 (83)	54.3 (25)*	1.6	20.7	6.3	0.7
Middle East, North Africa (51)	56.9 (29)	66.7 (14)	33.3 (3)*	0	29.4	11.8	2.0
South Asia (247)	64.8 (160)	70.4 (69)	63.5 (33)	0	25.5	8.5	2.0
Sub-Saharan Africa (993)	54.2 (538)	53.7 (212)	46.7 (120)*	0.9	29.1	24.7	0.7
TOTAL % (n local)	62.0 (1125)	62.9 (463)·	54.3 (229)	0.7	27.1	21.5	2.1

Due to author’s having multiple affiliations, the total percentage may exceed 100. *P < 0.05. IF Impact Factor (article in a journal that has an Impact Factor). Totals exclude systematic reviews and modelling studies, which cannot be classified into study region

### Differences among countries

There was substantial variation in patterns of authorship between the 60 LMICs which had 5 or more papers (Table 3). Only a quarter [16] of these 60 countries had a local first author in more than 75 % of their articles. In 10 of the 60 countries, fewer than 25 % of publications had a local first author. When countries with fewer than five publications were grouped together, the proportion of papers with a local lead was below half.

**Table 3:** Capacity and ownership of maternal health research in low- and middle-income countries

Country	Total papers in country (N)	Total papers as lead author N	Percentage as lead author %	Lead in papers in IF journals % (n)	Lead in papers in journal with IF ≥2 % (n)	Lead author has multiple affiliations %
Brazil	134	124	92.5	91.8 (45)	76.9 (10)*	1.5
South Africa	155	116	74.8	73.6 (53)	67.3 (33)	1.9
India	114	78	68.4	77.5 (31)	71.4 (15)	3.5
Nigeria	83	73	88.0	86.7 (26)	50.0 (3)*	2.4
Thailand	82	66	80.5	77.4 (24)	73.9 (17)	25.6
Kenya	99	43	43.4	36.8 (14)	29.0 (9)**	3.0
China	56	43	76.8	80.0 (28)	80.0 (16)	1.8
Tanzania	112	41	36.6	23.3 (10)	16.1 (5)*	15.2
Bangladesh	67	41	61.2	61.5 (16)	42.9 (6)	1.5
Uganda	82	39	47.6	47.4 (18)	40.7 (11)	11.0
Turkey	37	36	97.3	95.5 (21)	90.0 (9)**	0
Malawi	79	33	41.8	46.9 (15)	39.1 (9)	12.7
Côte d’Ivoire	48	25	52.1	43.8 (7)	38.5 (5)	29.2
Mexico	36	22	61.1	66.7 (8)	62.5 (5)	2.8
Zambia	49	21	42.9	37.0 (10)	36.4 (8)	26.5
Zimbabwe	39	21	53.8	56.3 (9)	58.3 (7)	15.4
Pakistan	30	20	66.7	92.3 (12)	100.0 (8)*	3.3
Burkina Faso	48	18	37.5	36.8 (7)	33.3 (5)	6.3
Ghana	56	17	30.4	20.0 (4)	25.0 (3)	3.6
Ethiopia	32	16	50.0	58.3 (7)	75.0 (3)	9.4
Nepal	31	16	51.6	50.0 (6)	33.3 (2)	0
Argentina	22	15	68.2	55.6 (5)	50.0 (3)	0
Cameroon	20	15	75.0	81.8 (9)	66.7 (4)	5.0
Sudan	19	15	78.9	100.0 (5)	100.0 (1)	5.3
Colombia	15	15	100.0	100 (5)	100 (1)	0
Egypt	25	11	44.0	53.9 (7)	0 (0)*	0
Botswana	24	11	45.8	40.0 (4)	40.0 (4)	37.5
Iran	11	10	90.9	75.0 (3)	0.0 (0)*	0
Mozambique	31	9	29.0	40.0 (4)	40.0 (4)	9.7
Rwanda	16	9	56.3	62.5 (5)	83.3 (5)**	12.5
Vietnam	22	8	36.4	22.2 (2)	16.7 (1)	9.1
Jamaica	13	8	61.5	66.7 (4)	50.0 (1)	7.7
Antigua and Barbuda	11	7	63.6	100.0 (4)	100.0 (2)	9.1
Chile	9	7	77.8	66.7 (2)	50.0 (1)	0
Panama	7	7	100.0	100 (2)	-	0
Uruguay	7	7	100.0	100 (3)	100 (1)	0
Mali	16	6	37.5	25.0 (2)	0.0 (0)*	0
Malaysia	7	6	85.7	80.0 (4)	50.0 (1)**	0
Indonesia	22	5	22.7	30.0 (3)	25.0 (2)	4.5
Peru	14	5	35.7	42.9 (3)	50.0 (3)	14.3
Haiti	6	5	83.3	100 (2)	100 (2)	50.0
The Gambia	6	5	83.3	100.0 (4)	100.0 (3)	16.7
Sri Lanka	5	5	100.0	100 (4)	100 (2)	20.0
Cambodia	11	4	36.4	25.0 (1)	0 (0)	9.1
Ecuador	9	4	44.4	50.0 (3)	33.3 (1)	0
Guatemala	15	3	20.0	28.6 (2)	33.3 (1)	0
Benin	14	3	21.4	50.0 (2)	50.0 (2)**	7.1
Philippines	8	3	37.5	50.0 (3)	75.0 (3)*	0.0
Madagascar	6	3	50.0	33.3 (1)	-	16.7
Myanmar	5	3	60.0	50.0 (1)	50.0 (1)	0
Dominican Republic	5	3	60.0	50.0 (1)	100.0 (1)	40.0
Bolivia	10	2	20.0	20.0 (1)	25.0 (1)	10.0
Senegal	10	2	20.0	25.0 (1)	0 (0)	10.0
Democratic Republic of the Congo	7	2	28.6	-	-	0
Niger	5	2	40.0	50.0 (1)	50.0 (1)	0
Honduras	8	1	12.5	25.0 (1)	33.3 (1)	12.5
Ukraine	7	1	14.3	0.0 (0)	0.0 (0)	0
Angola	7	1	14.3	0 (0)	0 (0)	0
Nicaragua	7	1	14.3	0 (0)	0 (0)	14.3
Russian Federation	5	0	0.0	0 (0)	0 (0)	0
Countries with fewer than 5 papers						
Countries with 3 papers^b^	27	10	37.0	-	-	3.7
Countries with 4 papers^a^	16	7	43.8	-	-	25.0
Countries with 1 paper^d^	19	7	36.8	-	-	5.3
Countries with 2 papers^c^	14	4	28.6	-	-	0

Countries ordered by total number of articles as first author.*P < 0.05. **P = 0.05–0.1, Chi-square test comparing the percentage with a local lead author in journals with Impact Factor ≥2 and <2.^a^Afghanistan, Gabon, Mongolia, Morocco.^b^Eritrea, Guinea, Lebanon, Sierra Leone, Somalia, Swaziland, Timor-Leste, Venezuela, Yemen.Armenia.^c^Cuba, Egypt, Georgia, Liberia, Republic of the Congo, West Bank.^d^Albania, Belarus, Bosnia Herzegovina, Chad, Fiji, Guinea Bissau, Guyana, Jordan, Kazakhstan, Lesotho, Mauritania, Moldova, Papua New Guinea, Paraguay, Republic of Macedonia, Romania, Solomon Islands, Syria, Tunisia. *IF* Impact Factor (article in a journal that has an Impact Factor)

**Fig. 3:**
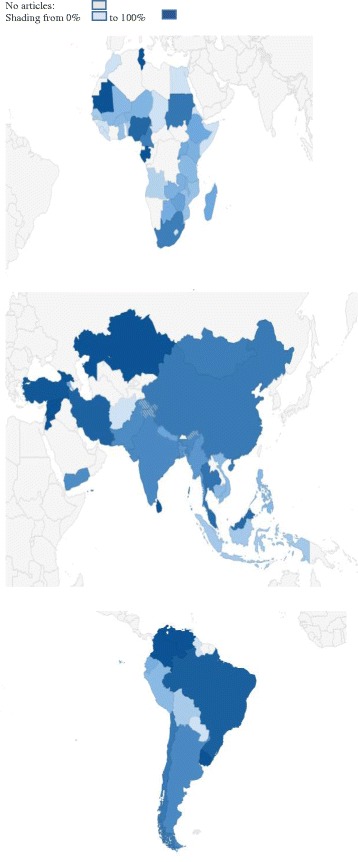
Percentage of local authors in LMIC publications on maternal health intervention studies

In four countries, *all* the papers were locally led; namely, Colombia (15 papers), Panama (7 papers), Uruguay (7 papers) and Sri Lanka (5 papers; Fig. 3). Almost all studies in Turkey had a local lead (36/37), and none of these authors had another affiliation outside Turkey.

**Fig. 4:**
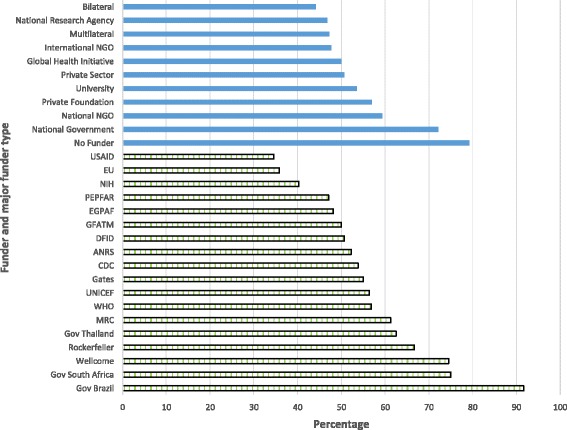
Proportion of locally-led articles by funder type and for the major funders. Note that Fig. 4 only includes individual funders with ≥40 papers and the three largest LMIC government funders

Nine LMICs had a lead author on 40 or more articles. In Brazil, the odds of having a local lead rose, on average, 2.4 fold with each of the three time periods (95 % CI = 0.94–6.2; *P* = 0.066), from 84.2 % in the period 2000–2002 (16/19) to 91.8 % in 2003–2007 (45/49) and finally 96.6 % in 2008–2012 (57/59). Though the number of publications rose considerably in India, the proportion of publications with a local lead declined from 91.7 % in 2000–2003 (11/12) to 66.0 % in 2004–2012 (66/100; *P* = 0.07). Trends in authorship patterns were noted in Mozambique and Ethiopia, though the confidence interval for these estimates was wide. With each of the three time periods, local authorship was 0.37 fold lower in Mozambique (95 % CI = 0.11–1.24; *P* = 0.11). Conversely, odds of local authorship were 2.08 fold higher in Ethiopia over each time period (95 % CI = 0.79–5.48; *P* = 0.14).

### Characteristics of HIC authors and authors with multiple affiliations

Authors affiliated with institutions in the United States (561 papers) and United Kingdom (207 papers) together account for a third of all articles (33.5 %), and mainland Europe for a further 21.5 % (452 papers; Table 4). Authors at institutions in mainland Europe, however, were 4.2 fold more likely to have multiple affiliations than those in the United States and United Kingdom (95 % CI OR = 2.8–6.1; *P* < 0.001). Only six LMIC authors had an affiliation in more than one LMIC, while 159 held both an LMIC and HIC affiliation. More than a quarter of lead authors in Botswana, Côte d’Ivoire, Thailand and Zambia had dual affiliations.

**Table 4:** High-income countries lead authorship of studies set in low- and middle-income countries

HIC country	Papers as lead author N	Proportion of papers in IF journal %	Proportion of papers in journal with IF ≥2 %	Dual affiliation with country of study % papers	Lead author has dual affiliation % papers
United States	561	40.6	31.4	8.0	10.3
United Kingdom	207	45.4	35.4	13.5	15.5
France	71	31.0	26.8	32.4	35.2
Canada	53	60.4	41.5	3.8	5.7
Netherlands	34	52.9	44.1	29.4	29.4
Australia	34	41.1	23.5	11.8	11.8
Sweden	33	33.3	9.1	18.2	30.3
Switzerland	29	34.5	34.5	3.4	6.9
Norway	25	32.0	20.0	24.0	28
Belgium	23	34.8	30.4	21.7	21.7
Germany	22	40.9	22.7	13.6	18.2
Italy	18	38.9	22.2	16.7	16.7
Denmark	11	72.7	36.4	18.2	27.3
Japan	7	42.9	28.6	0.0	0
Spain	6	33.3	33.3	16.7	33.3
Saudi Arabia	3	-	-	0.0	33.3
Austria	2	-	-	50.0	50
Finland	2	-	-	50.0	50
Ireland	2	-	-	0.0	0
New Zealand	2	-	-	0.0	0
Portugal	2	-	-	0.0	0
Scotland	2	-	-	0.0	0
Croatia	1	-	-	0.0	0
Greece	1	-	-	0.0	0
Singapore	1	-	-	0.0	0
TOTAL n (%)	1138	469 (41.65)	354 (31.1)	139 (12.2)	159 (14.0)

*IF* Impact Factor (article in a journal that has an Impact Factor). The proportion with IF is only shown for countries with ≥5 papers

HIC authors most commonly addressed health systems topics (39.0 %), followed by HIV (36.5 %). LMIC authors led a similar proportion of HIV (33.2 %) and health systems papers (32.6 %). HIV was the most common topic led by authors from institutions in the United States, France, Norway, Germany and Italy (Table 5). By contrast, the United Kingdom had a substantially larger focus on health systems research (46.4 % of their outputs) and malaria (19.6 %). A high proportion of studies led by the Netherlands addressed malaria in a maternal health population (35.3 %). Of studies led by a HIC author, 8.8 % were multi-country studies, compared with only 5.8 % of LMIC led papers (*P* = 0.006).

**Table 5:** Patterns of high-income authorship of maternal health interventional research in LMICs 2000–2012

Variable and categories	USA *N* = 561	UK *N* = 209	France *N* = 71	Canada *N* = 53	Netherland *N* = 34	Australia *N* = 34	Sweden *N* = 33	Switzerland *N* = 29	Norway *N* = 25	Belgium *N* = 23	Germany *N* = 22	Italy *N* = 18	Denmark *N* = 11	Multi-affilat.*N* = 150	TOTAL HIC^b^ col %	TOTAL LMIC^b^ col %
% of all publications in the study	24.5	9.1	3.1	2.3	1.5	1.5	1.4	1.3	1.1	1	1	0.8	0.5	6.5	50.3	49.7
% of all HIC-led publications in study	49.3	18.4	6.2	4.7	3	3	2.9	2.5	2.2	2	1.9	1.6	1	12.6	-	-
Economic region of research^a^ (col % of research excluding systematic reviews and formative research)																
UMIC	21.6	23.3	20	14.8	15	18.2	12.1	62.5	28.6	4.8	10.5	0	0	26.7	20.3	47.2*
LMIC	35	34	60	44.4	15	50	37.5	43.8	0	14.3	26.3	7.7	40	26.0	35.0	28.4*
LIC	49.8	50.5	33.3	44.4	70	36.4	56.3	56.3	76.2	81	63.2	92.3	60	44.0	51.9	25.9*
Geographic region of research^a^ (col % of research excluding systematic reviews and formative research)																
East Asia Pacific	8.1	14.8	14.5	6.7	15	62.5	6.1	11.3	0	8.7	5.3	0	10	18.7	11.8	11.6
Europe, Central Asia	1.5	4.3	0	3.3	0	0	3	3.2	4.8	0	0	0	20	0	2.1	4.3*
Latin America, Caribbean	13.3	7.8	3.2	6.7	0	0	0	16.2	0	0	0	0	0	5.3	10.5	21.8*
Middle East, N. Africa	3.3	0.9	0	0	0	0	0	2.7	0	4.3	5.3	0	0	0.7	2.7	2.9
South Asia	12.9	12.2	1.6	13.3	0	16.7	9.1	13.2	0	0	5.3	0	0	2.7	11.1	15.6*
Sub-Saharan Africa	59.3	54.8	79.0	60	85	12.5	78.8	52.9	95.2	78.3	84.2	100	70	67.3	67.2	44.7*
Time periods (col % of all research by HIC)																
2000–2003	18.3	19.4	14.1	17	9.1	3	15.6	20.7	4	34.8	14.3	0	10	16.9	17.0	15.9
2004–2007	38.5	28.2	38	26.4	12.1	24.2	28.1	31	36	13	38.1	64.7	50	35.1	33.9	34.6
2008–2012	43.1	52.4	47.9	56.6	78.8	72.7	56.3	48.3	60	52.2	47.6	35.3	40	48	49.1	49.6
Research topics (col % of all research on each topic)																
Haemorrhage	10.3	7.7	0	11.3	5.9	2.9	9.1	27.6	0	0	4.5	5.6	18.2	1.3	8.9	8.5
Hypertension	2.5	13.4	0	28.3	26.5	11.8	3	24.1	4	0	4.5	5.6	0	2.7	7.3	13.1*
HIV	42.4	14.8	81.7	20.8	23.5	5.9	18.2	31	44	43.5	36.4	88.9	27.3	54.7	36.5	33.2
STIs other than HIV	4.3	5.3	1.4	3.8	2.9	0	0	24.1	16	21.7	0	0	0	4	4.8	6.0
Malaria	9.8	19.6	15.5	3.8	35.3	14.7	6.1	6.9	0	17.4	27.3	0	18.2	25.3	12.6	12.3
Health Systems	40.8	46.4	8.5	32.1	20.6	52.9	57.6	31	40	56.5	22.7	11.1	36.4	26.0	39	32.6*
EmOC	12.1	10	2.8	17	2.9	5.9	18.2	10.3	16	8.7	0	0	27.3	4.7	10.9	7.1*
Demand-side Financing	1.8	6.7	1.4	3.8	0	8.8	6.1	0	0	13	9.1	0	0	2	3.4	2.8
Equity Examined	9.4	9.6	7	11.3	5.9	8.8	6.1	0	0	13	18.2	5.6	18.2	10.7	9.2	8.7
TBA	7.3	4.3	0	1.9	2.9	11.8	6.1	0	0	0	9.1	0	0	0	5.5	4.4
Main topic addressed	*HIV*	*Health System*	*HIV*	*Health System*	*Malaria*	*Health System*	*Health System*	*Health System*	*HIV*	*Health System*	*HIV*	*HIV*	*Health System*	*HIV*	*Health System*	*HIV*
Study design of research led by HIC (col %)*																
Systematic Review	7.8	33	7	37.7	32.4	29.4	0	20.7	8	0	4.5	16.7	9.1	1.6	15.6	5.8**
Effectiveness	60.2	40.7	62	34	44.1	44.1	69.7	24.1	60	82.6	59.1	72.2	45.5	7.8	54.5	68.3
RCT	14.6	7.7	16.9	13.2	0	2.9	9.1	34.5	4	13	9.1	0	27.3	6.6	12.5	13.9
Qualitative	3.9	2.9	5.6	5.7	5.9	17.6	15.2	0	12	0	9.1	0	0	4.1	4.7	6.0
Formative	10.7	12	5.6	5.7	8.8	0	0	17.2	8	0	9.1	11.1	0	4.1	9.1	3.9
Mixed Methods	2.7	3.8	2.8	3.8	8.8	5.9	6.1	3.4	8	4.3	9.1	0	18.2	7.5	3.7	2.2
Multi-country studies (%)*																
Multi-country study	8.4	9.6	12.7	5.7	11.8	2.9	9.1	34.5	4	4.3	0	11.1	0	6	8.8	5.8*

Due to multi-country studies, total percentage may exceed 100. ^a^Proportions may sum to below 100 % as papers on systematic reviews and modelling were not classified by economic and geographical region. Chi-square test comparing the percentage with a high-income country lead author to local leads. *P < 0.05 for each sub-category of multiple response variables. **P <0.05, tests for association between HIC and LMIC in mutually exclusive categorical variable, such as study design. ^b^ HIC totals include all papers with a HIC author (including those with a dual HIC and LMIC affiliation). LMIC totals include only papers where the author has only an LMIC affiliation (excluding those with a dual LMIC and HIC affiliation

### Influence of maternal health topic, study design and research funder

More than 80 % of hypertension studies had a locally-affiliated first author, compared with only 54.3 % of haemorrhage and 59.2 % of HIV studies. LMIC authors were 1.92 fold more likely to lead papers on hypertension compared with their HIC counterparts (95 % CI = 1.45–2.55; *P* < 0.001). Around half of articles covering a health systems intervention for maternal health had a local author (51.4 %). The proportion of malaria articles with a local first author declined stepwise over time, from 82.4 % in 2000–2003 (28/34) to 69.2 % in 2004–2007 (54/78) and 61.7 % in 2008–2012 (74/120, *P* = 0.03).

Overall, 58.7 % of randomised controlled trials (RCTs) had a local lead (175/298), with little difference if authors with multiple affiliations were excluded (56.5 %, 157/278). In sub-Saharan Africa, 48.3 % of RCTs overall had a local first author (86/178), while this figure was only 35.2 % for RCTs on HIV in the region (32/91). Notably, HIC authors dominated research that applied mixed methods (61.2 %, 41/67), systematic review (73.3 %; 178/243) or modelling techniques (70.1 %, 103/147).

Funding agencies that are characterised by collaborations between an individual HIC and LMIC (bilateral and HIC national research agencies) had fewer locally-led articles than private or global funders (Fig. 4). Of note, even among studies that acknowledged funding by national governments in LMICs, only 72.1 % were locally led (44/61), while 79.2 % of studies with no funder acknowledgement had a local lead (454/573). Of individual funders (as opposed to types of funding agencies, such as bilateral or national research agencies), HIC authors dominated the outputs of research involving United States Agency for International Development (65.5 %, 110/168), the European Union (64.2 %, 34/53) and the National Institutes of Health (59.7 %, 126/211). Compared to these three funders, studies supported by the Wellcome Trust and The Rockefeller Foundation, had considerably fewer HIC leads (25.5 %, 14/55; and 33.3 %, 4/12 respectively).

### Variation by journal Impact Factor

Just above forty percent of papers were published in a journal with an Impact Factor, with almost identical proportions for LMIC (41.7 %; 469/1126) and HIC (41.6; 473/1138) affiliated authors. Of note, authors affiliated with an LMIC institution led 68.2 % of articles in journals with an Impact Factor below 2 (255/374), 39.8 % of those in journals with Impact Factor 2–5 (153/384), and only 33.2 % of articles in a journal with an Impact Factor above 5 (61/184; *P* < 0.001). Importantly, the median Impact Factor was 3.1 (IQR = 1.9–5.3) for HIC first authors and 1.8 (IQR = 1.1–3.3) for those in LMICs (*P* < 0.001).

Brazil and Kenya provide illustrative examples of how researchers in HIC institutions dominate publications in high Impact Factor journals. Authors with foreign affiliations led only 7.5 % of all articles in Brazil (10/134), but 23.1 % of papers in journals with an Impact Factor above 2 (*P* = 0.02). Similarly, in Kenya, HIC authors accounted for 56.6 % of all articles (56/99), but an even higher proportion of those in journals with an Impact Factor above 2 (71.0 %, 22/31; *P* = 0.05). Above 40 % of articles led by authors affiliated with institutions in the Netherlands (15/44) and Canada (22/53) were published in journals with an Impact Factor above 2 (Table 4).

## Discussion

The study details marked deficiencies in lead authorship in many countries and on several research topics. This suggests deficits in local ownership of data in the field of maternal health interventional research within LMICs, as well as in the capacity to analyse and articulate research findings. The figure of about 60 % of research on maternal health interventions in LMICs being led by authors affiliated with an LMIC institution is higher than research in the fields of tropical medicine [1] and international epidemiology [8], but much lower than in studies of orthopaedics [9] or tuberculosis in LMICs [10]. In our study, it is striking that HIC researchers lead two thirds of articles in journals with an Impact Factor above 5, and even higher proportions of systematic review and modelling studies. The capacity of countries to advance their own maternal health research agendas appears to vary markedly: only a quarter of countries led more than 75 % of their research, while another quarter led under 25 %. Aside from Brazil and the Middle East, North Africa region, no other countries or geographical regions increased the proportion of articles led over time. Finally, the rate of increase in number of publications by LMIC authors diminished from 2008 onwards. This might indicate a change in funding patterns for maternal health, different pressures to publish on HIC country researchers, or perhaps that many LMIC institutions had reached a ceiling in their capacity to publish findings of maternal health research.

The study demonstrates the presence of many North–south research collaborations in maternal health. North–south collaborations take many forms, including the partnering of northern and southern institutions [25], who then write joint research proposals, share mentoring of post-graduate students [26] and have staff exchanges, for example. Other initiatives entail the development of international databases and consortia who generate and analyse large databases, such as in the International Epidemiologic Databases to Evaluate AIDS (IeDEA) network [27]; and specific efforts to strengthen capacity in southern countries, such as in research ethics [28]. In contrast with well-developed North–south relations, the negligible number of LMIC authors who hold affiliations or lead work in another LMIC, suggests that South-South partnerships in maternal health are poorly developed [3, 29]. Genuine research partnerships are clearly important for creating and sustaining research in LMICs; through offering efficiencies by the transfer of technologies between countries, economies of scale and productivity, and broadening the visibility of a project [30]. The concept of research partnerships and authorship practices within such partnerships seemingly varies considerably, as illustrated by the marked discrepancies in authorship practices across funders and HICs [31, 32]. Interestingly, several large global malaria partnerships began around 2000 (see, for example, [33, 34]), likely accounting for the rise in number of publications on malaria. This was, however, accompanied by a drop in the proportion of locally led articles on the topic, possibly indicating that such partnerships might undermine data ownership. Conversely, hypertension publications are predominately locally led, but the total number of these papers rose only slightly over time, and we could not identify any high-profile global partnerships focused on hypertension in pregnancy.

Present internationally-agreed authorship criteria provide little, if any, recognition for the ‘technical tasks’ of research, even though these require considerable scientific skills and expertise [12]. The funding that LMIC researchers receive from international research partnerships is predominantly for these ‘technical tasks’ (such as running clinical research sites, participant recruitment and retention, community partnership and local dissemination), rather than for developing their own research ideas, analysing data and completing publications. Also, the dominance of United States and United Kingdom authors may partly be explained by the substantial linguistic challenges faced by non-Anglophone authors when drafting and critically revising an article in English [35]. These considerations might explain why fewer locally-led studies in Latin America were published within journals with a high Impact Factor. Finally, local researchers may even prefer a better-known HIC counterpart to be the first author, in the belief that this raises prospects of publication and future citation [8].

Journals themselves have made some efforts to address the above concerns, including waiving publication fees for LMIC authors, devoting space to local research from specific regions and providing editing assistance to non-English speakers [35]. In a similar spirit, research collaborations might adopt strategies such as alternating first authorship between HIC and LMIC researchers, or publishing as a collective. The intent of such initiatives is, in part, to redress capacity deficits or past discrimination, and to place LMIC and HIC researchers on a more equal footing.

## Study limitations and research gaps

The methods employed in this research extend other bibliometric means of measuring authorship of publications [29, 30], by using data from full text articles to investigate a broader range of factors that might explain authorship patterns. While the indicator ‘lead author’ provides a proxy measure of data ownership and research leadership, assessing affiliations of all authors of an article would have allowed for a more comprehensive evaluation. Authors with multiple affiliations were categorised as being locally affiliated if they held an affiliation in the country of study, and the direction of bias introduced by this classification is hard to ascertain. It is likely that the authorship practices and research contributions of researchers holding a dual HIC and LMIC affiliation vary considerably, making it difficult to interpret this variable. Researchers, from LMICs and HICs alike, may increasingly be highly mobile and affiliations shift rapidly over time. It is possible, for example, that LMIC nationals live and conduct research in their country, while holding an affiliation in a HIC. Classifying those individuals as HIC authors would mean that the proxy measure ‘lead author’ used in this study underestimates the actual research capacity of LMIC countries. As a further limitation, the study only measures one component of research capacity (ability to analyse and articulate findings), and cannot comment on the other elements thereof. Furthermore, importantly, the number of articles is a reflection of the level of external funding for a topic, not only of data ownership and research capacity. The analysis also does not assess whether a relatively small group of authors at LMIC institutions account for most papers in an LMIC [29], which might explain the slowing of increases in number of papers, as research institutes reach a maximum size. Finally, assessments, similar to the one presented here, repeated over time, might serve to monitor changing trends in scientific capacity and data ownership of LMICs [14].

## Conclusion

Health challenges in LMICs remain grievous, as again demonstrated by the Ebola outbreak. Substantial advances were made in the number of LMIC lead authorships in the mid 2000s, but increments slowed thereafter. This suggests that different approaches might now be required to achieve further gains in research capacity; with approaches tailored to the specific constraints in different settings. Steps taken might include mentorship on leading complex research projects, improved research infrastructure and specific funding for academic writing [36]. Increased investments by LMIC governments for strengthening research capacity at all levels may also further raise research skills and infrastructure.

The proxy for data ownership, the proportion of articles led by authors affiliated to LMIC institutions, rose slowly, if at all, in most categories assessed. Gaps in systematic review expertise in LMICs are especially concerning [3], given the importance of these skills for evaluating and synthesizing locally-relevant evidence to inform policy decisions. South-South collaborations appear weak, with few joint LMIC cross-country authorships or joint affiliations identified. This might reflect limited funding for South-South work.

More stringent oversight of authorship attribution may accelerate gains made in local authorship and counter any residual inequities between LMIC researchers on the one hand, and their more prominent HIC counterparts or funding agencies on the other. Revision of international authorship guidelines (e.g. ICMJE [37]) may be warranted, with specific recommendations to guide authorship attribution between LMIC and HIC researchers. For example, for papers where the first author is not affiliated with an institution in the country of study, journals could require authors to state why it was not possible for a local author to lead the paper. Already, some journals do not publish articles that do not include an author from the study country [38]. This issue has also gained political traction among policy leaders in Africa, such as the Rwanda health minister Agnes Binagwaho, who have argued that no research or article about a low-income country should be published without joint authorship from that country [39]. If not addressed, continued low levels of LMIC authorship may demoralise the increasingly experienced local researchers in LMICs and undermine global health research collaborations in this vital field of work. More importantly, stronger research leadership in LMICs will help ensure that a country’s research agenda is aligned with its knowledge needs, and not merely with the research interests of HIC scientists. Non-alignment can have substantial negative impacts on population health.

## Abbreviations

HIC, high-income country; ICMJE, International Committee of Medical Journal Editors; IQR, inter-quartile range; LMIC, lower middle income country

## Additional file

Additional file 1: Search strategy for review. (DOCX 25 kb)
