# Supplementary material for: Local and foreign authorship of maternal health interventional research in low- and middle-income countries: systematic mapping of publications 2000–2012
Source: Global Health. 2016 Jun 23;12:35. doi: 10.1186/s12992-016-0172-x (PMC4917998; doi:10.1186/s12992-016-0172-x)
Supplement: Additional file 1: — Search strategy for review. (DOCX 25 kb) [file 12992_2016_172_MOESM1_ESM.docx]

# Additional file 1: Search strategy for review

## PubMED search strategy

(((((non-pregnancy[All Fields] AND related[All Fields] AND ("infection"[MeSH Terms] OR "infection"[All Fields] OR "communicable diseases"[MeSH Terms] OR ("communicable"[All Fields] AND "diseases"[All Fields]) OR "communicable diseases"[All Fields])) OR non-pregnancy related[Title]) OR ((maternal[Title] OR pregnant[Title] OR pregnancy[Title] OR obstetric[Title] OR puerperal[Title] OR mother[Title] OR childbirth[Title] OR labour[Title] OR labor[Title] OR natal[Title] OR post-natal[Title] OR pre-natal[Title] OR prenatal[Title] OR antenatal[Title] OR ante-natal[Title] OR perinatal[Title] OR peri-natal[Title] OR puerperal[Title] OR puerperium[Title]) AND ((((((sepsis[Title] OR septic$[Title]) OR infection$[Title]) OR HIV[Title]) OR tuberculosis[Title]) OR pneumonia[Title]) OR meningitis[Title]))) OR (chorioamnionitis[Title/Abstract] OR "chorioamnionitis"[MeSH Terms])) OR ((("sepsis"[MeSH Terms] OR "sepsis"[All Fields]) OR septic$[All Fields] OR infection$[Title]) AND ((amniotic[Title/Abstract] OR intra-amniotic[Title/Abstract]) OR intraamniotic[Title/Abstract])))) OR ((((anemic[Title] OR anaemia[Title]) OR anaemic[Title]) OR anemia[Title]) AND (puerperal[Title] OR (((((maternal[Title] OR pregnant[Title]) OR pregnancy[Title]) OR obstetric[Title]) OR mother[Title]) OR childbirth[Title])))) OR (((((("Midwifery"[Mesh] OR dula[Title/Abstract]) OR ((("parturition"[MeSH Terms] OR "parturition"[All Fields] OR "birth"[All Fields]) AND attendant[All Fields]) OR (("parturition"[MeSH Terms] OR "parturition"[All Fields] OR "birth"[All Fields]) AND attendants[All Fields]))) OR ("residence characteristics"[MeSH Terms] OR ("residence"[All Fields] AND "characteristics"[All Fields]) OR "residence characteristics"[All Fields] OR ("place"[All Fields] AND "birth"[All Fields]) OR "place of birth"[All Fields])) OR (("Birthing Centers"[MAJR] OR "Delivery Rooms"[MAJR]) OR "Delivery, Obstetric/nursing"[MAJR])) OR ((maternal[Title] OR pregnant[Title] OR pregnancy[Title] OR obstetric[Title] OR puerperal[Title] OR mother[Title] OR childbirth[Title] OR labour[Title] OR labor[Title] OR natal[Title] OR post-natal[Title] OR pre-natal[Title] OR prenatal[Title] OR antenatal[Title] OR ante-natal[Title] OR perinatal[Title] OR peri-natal[Title] OR puerperal[Title] OR puerperium[Title]) AND (("Ambulances"[Mesh] OR "Health Services Accessibility"[Mesh]) OR "Transportation of Patients"[Mesh]))) OR (("Travel"[MeSH Terms] OR "Delivery of Health Care/organization and administration"[MAJR]) AND (maternal[Title] OR pregnant[Title] OR pregnancy[Title] OR obstetric[Title] OR puerperal[Title] OR mother[Title] OR childbirth[Title] OR labour[Title] OR labor[Title] OR natal[Title] OR post-natal[Title] OR pre-natal[Title] OR prenatal[Title] OR antenatal[Title] OR ante-natal[Title] OR perinatal[Title] OR peri-natal[Title] OR puerperal[Title] OR puerperium[Title])))) OR (ectopic pregnancy[Title/Abstract] OR "pregnancy, ectopic"[MeSH Terms])) OR (((((("Postpartum Hemorrhage"[Mesh] OR (((((((((((((((((maternal[Title] OR pregnant[Title]) OR pregnancy[Title]) OR obstetric[Title]) OR puerperal[Title]) OR mother[Title]) OR childbirth[Title]) OR labour[Title]) OR labor[Title]) OR natal[Title]) OR post-natal[Title]) OR pre-natal[Title]) OR prenatal[Title]) OR antenatal[Title]) OR ante-natal[Title]) OR perinatal[Title]) OR peri-natal[Title]) AND (Hemorrhage[Title] OR Haemorrhage[Title]))) OR (((obstetric[All Fields] AND ("haemorrhage"[All Fields] OR "hemorrhage"[MeSH Terms] OR "hemorrhage"[All Fields])) OR obstetric hemorrhage[Title/Abstract]) OR ("postpartum hemorrhage"[MeSH Terms] OR ("postpartum"[All Fields] AND "hemorrhage"[All Fields]) OR "postpartum hemorrhage"[All Fields] OR ("post"[All Fields] AND "partum"[All Fields] AND "hemorrhage"[All Fields]) OR "post partum hemorrhage"[All Fields]) OR ("postpartum hemorrhage"[MeSH Terms] OR ("postpartum"[All Fields] AND "hemorrhage"[All Fields]) OR "postpartum hemorrhage"[All Fields] OR ("post"[All Fields] AND "partum"[All Fields] AND "haemorrhage"[All Fields]) OR "post partum haemorrhage"[All Fields]) OR ("postpartum hemorrhage"[MeSH Terms] OR ("postpartum"[All Fields] AND "hemorrhage"[All Fields]) OR "postpartum hemorrhage"[All Fields] OR ("post"[All Fields] AND "partum"[All Fields] AND "hemorrhage"[All Fields]) OR "post partum hemorrhage"[All Fields]) OR ("postpartum hemorrhage"[MeSH Terms] OR ("postpartum"[All Fields] AND "hemorrhage"[All Fields]) OR "postpartum hemorrhage"[All Fields] OR ("post"[All Fields] AND "partum"[All Fields] AND "haemorrhage"[All Fields]) OR "post partum haemorrhage"[All Fields]))) OR obstetric hemorrhage[Title/Abstract]) OR "Hypertension, Pregnancy-Induced"[Mesh]) OR ((((obstructed labor[Title/Abstract] OR obstructed labour[Title/Abstract]) OR (obstetric fistula[Title/Abstract] OR obstetric fistulae[Title/Abstract])) OR ("vaginal fistula"[MeSH Terms] OR "vesicovaginal fistula"[MeSH Terms])) OR ("Obstetric Labor Complications"[Mesh] OR "Obstetric Labor, Premature"[Mesh]))) OR ((((((((((((((((((maternal[Title] OR pregnant[Title]) OR pregnancy[Title]) OR obstetric[Title]) OR puerperal[Title]) OR mother[Title]) OR childbirth[Title]) OR labour[Title]) OR labor[Title]) OR natal[Title]) OR post-natal[Title]) OR pre-natal[Title]) OR prenatal[Title]) OR antenatal[Title]) OR ante-natal[Title]) OR perinatal[Title]) OR peri-natal[Title]) AND (hypertension[Title] OR blood pressure[Title])) AND (((((eclampsia[Title/Abstract] OR preeclampsia[Title/Abstract]) OR HELLP[Title/Abstract]) OR "eclampsia"[MeSH Terms]) OR "pre-eclampsia"[MeSH Terms]) OR pre-eclampsia[Title/Abstract])))) OR ("Pregnancy Complications, Hematologic"[Mesh] OR "Pregnancy in Adolescence"[Mesh] OR "Pregnancy Complications, Infectious"[Mesh] OR "Pregnancy Complications, Cardiovascular"[Mesh] OR "Pregnancy Complications"[Mesh] OR "Pregnancy, Prolonged"[Mesh])) AND (((("africa"[MeSH Terms] OR "africa"[All Fields]) OR (((((((("afghanistan"[MeSH Terms] OR "afghanistan"[All Fields]) OR ("bangladesh"[MeSH Terms] OR "bangladesh"[All Fields])) OR ("benin"[MeSH Terms] OR "benin"[All Fields])) OR ("burkina faso"[MeSH Terms] OR ("burkina"[All Fields] AND "faso"[All Fields]) OR "burkina faso"[All Fields])) OR (((((((((((((((((((((((((((("burundi"[MeSH Terms] OR "burundi"[All Fields]) OR ("cambodia"[MeSH Terms] OR "cambodia"[All Fields])) OR ("central african republic"[MeSH Terms] OR ("central"[All Fields] AND "african"[All Fields] AND "republic"[All Fields]) OR "central african republic"[All Fields])) OR ("chad"[MeSH Terms] OR "chad"[All Fields])) OR ("comoros"[MeSH Terms] OR "comoros"[All Fields])) OR (("congo"[MeSH Terms] OR "congo"[All Fields]) AND Dem.[All Fields] AND Rep[All Fields])) OR ("congo"[MeSH Terms] OR "congo"[All Fields])) OR DRC[Affiliation]) OR ("eritrea"[MeSH Terms] OR "eritrea"[All Fields])) OR ("ethiopia"[MeSH Terms] OR "ethiopia"[All Fields])) OR ("gambia"[MeSH Terms] OR "gambia"[All Fields])) OR ("guinea"[MeSH Terms] OR "guinea"[All Fields])) OR (("guinea"[MeSH Terms] OR "guinea"[All Fields]) AND Bisau[All Fields])) OR ("haiti"[MeSH Terms] OR "haiti"[All Fields])) OR ("kenya"[MeSH Terms] OR "kenya"[All Fields])) OR ("korea"[MeSH Terms] OR "korea"[All Fields])) OR Kyrgyz[All Fields]) OR ("liberia"[MeSH Terms] OR "liberia"[All Fields])) OR ("madagascar"[MeSH Terms] OR "madagascar"[All Fields])) OR ("malawi"[MeSH Terms] OR "malawi"[All Fields])) OR ("mali"[MeSH Terms] OR "mali"[All Fields])) OR ("mozambique"[MeSH Terms] OR "mozambique"[All Fields])) OR ("myanmar"[MeSH Terms] OR "myanmar"[All Fields])) OR ("nepal"[MeSH Terms] OR "nepal"[All Fields])) OR ("niger"[MeSH Terms] OR "niger"[All Fields])) OR ("rwanda"[MeSH Terms] OR "rwanda"[All Fields])) OR ("sierra leone"[MeSH Terms] OR ("sierra"[All Fields] AND "leone"[All Fields]) OR "sierra leone"[All Fields])) OR ("somalia"[MeSH Terms] OR "somalia"[All Fields]))) OR ((((("tajikistan"[MeSH Terms] OR "tajikistan"[All Fields]) OR ("tanzania"[MeSH Terms] OR "tanzania"[All Fields])) OR ("togo"[MeSH Terms] OR "togo"[All Fields])) OR ("uganda"[MeSH Terms] OR "uganda"[All Fields])) OR ("zimbabwe"[MeSH Terms] OR "zimbabwe"[All Fields]))) OR ("africa, northern"[MeSH Terms] OR ("africa"[All Fields] AND "northern"[All Fields]) OR "northern africa"[All Fields] OR "sahara"[All Fields])) OR sub-saharan[All Fields])) OR ((((((((((((((((((((((((((((((((((((((((((((((((((((((((((((("angola"[MeSH Terms] OR "angola"[All Fields]) OR ("armenia"[MeSH Terms] OR "armenia"[All Fields])) OR ("belize"[MeSH Terms] OR "belize"[All Fields])) OR ("bhutan"[MeSH Terms] OR "bhutan"[All Fields])) OR ("bolivia"[MeSH Terms] OR "bolivia"[All Fields])) OR ("cameroon"[MeSH Terms] OR "cameroon"[All Fields])) OR ("cape verde"[MeSH Terms] OR ("cape"[All Fields] AND "verde"[All Fields]) OR "cape verde"[All Fields])) OR ("congo"[MeSH Terms] OR "congo"[All Fields])) OR ("cote d'ivoire"[MeSH Terms] OR ("cote"[All Fields] AND "d'ivoire"[All Fields]) OR "cote d'ivoire"[All Fields])) OR ("cote d'ivoire"[MeSH Terms] OR ("cote"[All Fields] AND "d'ivoire"[All Fields]) OR "cote d'ivoire"[All Fields] OR ("ivory"[All Fields] AND "coast"[All Fields]) OR "ivory coast"[All Fields])) OR ("djibouti"[MeSH Terms] OR "djibouti"[All Fields])) OR ("egypt"[MeSH Terms] OR "egypt"[All Fields])) OR ("el salvador"[MeSH Terms] OR ("el"[All Fields] AND "salvador"[All Fields]) OR "el salvador"[All Fields])) OR ("fiji"[MeSH Terms] OR "fiji"[All Fields])) OR ("georgia"[MeSH Terms] OR "georgia"[All Fields] OR "georgia (republic)"[MeSH Terms] OR ("georgia"[All Fields] AND "(republic)"[All Fields]) OR "georgia (republic)"[All Fields])) OR ("ghana"[MeSH Terms] OR "ghana"[All Fields])) OR ("guatemala"[MeSH Terms] OR "guatemala"[All Fields])) OR ("guyana"[MeSH Terms] OR "guyana"[All Fields])) OR ("honduras"[MeSH Terms] OR "honduras"[All Fields])) OR ("indonesia"[MeSH Terms] OR "indonesia"[All Fields])) OR ("india"[MeSH Terms] OR "india"[All Fields])) OR ("iraq"[MeSH Terms] OR "iraq"[All Fields])) OR ("micronesia"[MeSH Terms] OR "micronesia"[All Fields] OR "kiribati"[All Fields])) OR ("yugoslavia"[MeSH Terms] OR "yugoslavia"[All Fields] OR "kosovo"[All Fields])) OR Lao[All Fields]) OR ("lesotho"[MeSH Terms] OR "lesotho"[All Fields])) OR ("micronesia"[MeSH Terms] OR "micronesia"[All Fields] OR ("marshall"[All Fields] AND "islands"[All Fields]) OR "marshall islands"[All Fields])) OR ("mauritania"[MeSH Terms] OR "mauritania"[All Fields])) OR ("micronesia"[MeSH Terms] OR "micronesia"[All Fields])) OR ("moldova"[MeSH Terms] OR "moldova"[All Fields])) OR ("mongolia"[MeSH Terms] OR "mongolia"[All Fields])) OR ("morocco"[MeSH Terms] OR "morocco"[All Fields])) OR ("nicaragua"[MeSH Terms] OR "nicaragua"[All Fields])) OR ("nigeria"[MeSH Terms] OR "nigeria"[All Fields])) OR ("pakistan"[MeSH Terms] OR "pakistan"[All Fields])) OR ("papua new guinea"[MeSH Terms] OR ("papua"[All Fields] AND "new"[All Fields] AND "guinea"[All Fields]) OR "papua new guinea"[All Fields])) OR ("paraguay"[MeSH Terms] OR "paraguay"[All Fields])) OR ("philippines"[MeSH Terms] OR "philippines"[All Fields])) OR ("samoa"[MeSH Terms] OR "samoa"[All Fields])) OR ("atlantic islands"[MeSH Terms] OR ("atlantic"[All Fields] AND "islands"[All Fields]) OR "atlantic islands"[All Fields] OR ("sao"[All Fields] AND "tome"[All Fields] AND "principe"[All Fields]) OR "sao tome and principe"[All Fields])) OR (Sao[All Fields] AND Tome[All Fields])) OR ("senegal"[MeSH Terms] OR "senegal"[All Fields])) OR ("melanesia"[MeSH Terms] OR "melanesia"[All Fields] OR ("solomon"[All Fields] AND "islands"[All Fields]) OR "solomon islands"[All Fields])) OR ("sri lanka"[MeSH Terms] OR ("sri"[All Fields] AND "lanka"[All Fields]) OR "sri lanka"[All Fields])) OR ("sudan"[MeSH Terms] OR "sudan"[All Fields])) OR ("swaziland"[MeSH Terms] OR "swaziland"[All Fields])) OR ("syria"[MeSH Terms] OR "syria"[All Fields] OR ("syrian"[All Fields] AND "arab"[All Fields] AND "republic"[All Fields]) OR "syrian arab republic"[All Fields])) OR ("syria"[MeSH Terms] OR "syria"[All Fields])) OR ("east timor"[MeSH Terms] OR ("east"[All Fields] AND "timor"[All Fields]) OR "east timor"[All Fields] OR ("timor"[All Fields] AND "leste"[All Fields]) OR "timor leste"[All Fields])) OR ("indonesia"[MeSH Terms] OR "indonesia"[All Fields] OR "timor"[All Fields])) OR ("tonga"[MeSH Terms] OR "tonga"[All Fields])) OR ("turkmenistan"[MeSH Terms] OR "turkmenistan"[All Fields])) OR ("micronesia"[MeSH Terms] OR "micronesia"[All Fields] OR "tuvalu"[All Fields])) OR ("ukraine"[MeSH Terms] OR "ukraine"[All Fields])) OR ("uzbekistan"[MeSH Terms] OR "uzbekistan"[All Fields])) OR ("vanuatu"[MeSH Terms] OR "vanuatu"[All Fields])) OR ("vietnam"[MeSH Terms] OR "vietnam"[All Fields])) OR (("middle east"[MeSH Terms] OR ("middle"[All Fields] AND "east"[All Fields]) OR "middle east"[All Fields] OR ("west"[All Fields] AND "bank"[All Fields]) OR "west bank"[All Fields]) AND Gaza[All Fields])) OR Gaza[All Fields]) OR ("yemen"[MeSH Terms] OR "yemen"[All Fields])) OR ("zambia"[MeSH Terms] OR "zambia"[All Fields]))) OR (((((((((((((((((((((((((((((((((((((((((((((((((((((((((((((((((((("albania"[MeSH Terms] OR "albania"[All Fields]) OR ("algeria"[MeSH Terms] OR "algeria"[All Fields])) OR ("american samoa"[MeSH Terms] OR ("american"[All Fields] AND "samoa"[All Fields]) OR "american samoa"[All Fields])) OR ("samoa"[MeSH Terms] OR "samoa"[All Fields])) OR ("antigua and barbuda"[MeSH Terms] OR ("antigua"[All Fields] AND "barbuda"[All Fields]) OR "antigua and barbuda"[All Fields])) OR ("antigua and barbuda"[MeSH Terms] OR ("antigua"[All Fields] AND "barbuda"[All Fields]) OR "antigua and barbuda"[All Fields] OR "antigua"[All Fields])) OR ("antigua and barbuda"[MeSH Terms] OR ("antigua"[All Fields] AND "barbuda"[All Fields]) OR "antigua and barbuda"[All Fields] OR "barbuda"[All Fields])) OR ("argentina"[MeSH Terms] OR "argentina"[All Fields])) OR ("azerbaijan"[MeSH Terms] OR "azerbaijan"[All Fields])) OR ("republic of belarus"[MeSH Terms] OR ("republic"[All Fields] AND "belarus"[All Fields]) OR "republic of belarus"[All Fields] OR "belarus"[All Fields])) OR ("bosnia-herzegovina"[MeSH Terms] OR "bosnia-herzegovina"[All Fields] OR ("bosnia"[All Fields] AND "herzegovina"[All Fields]) OR "bosnia and herzegovina"[All Fields])) OR ("bosnia-herzegovina"[MeSH Terms] OR "bosnia-herzegovina"[All Fields] OR "bosnia"[All Fields])) OR ("bosnia-herzegovina"[MeSH Terms] OR "bosnia-herzegovina"[All Fields] OR "herzegovina"[All Fields])) OR ("botswana"[MeSH Terms] OR "botswana"[All Fields])) OR ("brazil"[MeSH Terms] OR "brazil"[All Fields])) OR ("bulgaria"[MeSH Terms] OR "bulgaria"[All Fields])) OR ("chile"[MeSH Terms] OR "chile"[All Fields])) OR ("china"[MeSH Terms] OR "china"[All Fields])) OR ("colombia"[MeSH Terms] OR "colombia"[All Fields])) OR ("costa rica"[MeSH Terms] OR ("costa"[All Fields] AND "rica"[All Fields]) OR "costa rica"[All Fields])) OR ("cuba"[MeSH Terms] OR "cuba"[All Fields])) OR ("dominica"[MeSH Terms] OR "dominica"[All Fields])) OR ("dominican republic"[MeSH Terms] OR ("dominican"[All Fields] AND "republic"[All Fields]) OR "dominican republic"[All Fields])) OR ("ecuador"[MeSH Terms] OR "ecuador"[All Fields])) OR ("gabon"[MeSH Terms] OR "gabon"[All Fields])) OR ("grenada"[MeSH Terms] OR "grenada"[All Fields])) OR ("iran"[MeSH Terms] OR "iran"[All Fields])) OR ("jamaica"[MeSH Terms] OR "jamaica"[All Fields])) OR ("jordan"[MeSH Terms] OR "jordan"[All Fields])) OR ("kazakhstan"[MeSH Terms] OR "kazakhstan"[All Fields])) OR ("latvia"[MeSH Terms] OR "latvia"[All Fields])) OR ("lebanon"[MeSH Terms] OR "lebanon"[All Fields])) OR ("libya"[MeSH Terms] OR "libya"[All Fields])) OR ("lithuania"[MeSH Terms] OR "lithuania"[All Fields])) OR ("macedonia (republic)"[MeSH Terms] OR ("macedonia"[All Fields] AND "(republic)"[All Fields]) OR "macedonia (republic)"[All Fields] OR "macedonia"[All Fields])) OR ("malaysia"[MeSH Terms] OR "malaysia"[All Fields])) OR ("indian ocean islands"[MeSH Terms] OR ("indian"[All Fields] AND "ocean"[All Fields] AND "islands"[All Fields]) OR "indian ocean islands"[All Fields] OR "maldives"[All Fields])) OR ("mauritius"[MeSH Terms] OR "mauritius"[All Fields])) OR ("comoros"[MeSH Terms] OR "comoros"[All Fields] OR "mayotte"[All Fields])) OR ("mexico"[MeSH Terms] OR "mexico"[All Fields])) OR ("montenegro"[MeSH Terms] OR "montenegro"[All Fields])) OR ("namibia"[MeSH Terms] OR "namibia"[All Fields])) OR ("palau"[MeSH Terms] OR "palau"[All Fields])) OR ("panama"[MeSH Terms] OR "panama"[All Fields])) OR ("peru"[MeSH Terms] OR "peru"[All Fields])) OR ("romania"[MeSH Terms] OR "romania"[All Fields])) OR ("russia"[MeSH Terms] OR "russia"[All Fields] OR ("russian"[All Fields] AND "federation"[All Fields]) OR "russian federation"[All Fields])) OR ("russia"[MeSH Terms] OR "russia"[All Fields])) OR ("ussr"[MeSH Terms] OR "ussr"[All Fields])) OR ("serbia"[MeSH Terms] OR "serbia"[All Fields])) OR ("seychelles"[MeSH Terms] OR "seychelles"[All Fields])) OR ("south africa"[MeSH Terms] OR ("south"[All Fields] AND "africa"[All Fields]) OR "south africa"[All Fields])) OR ("saint kitts and nevis"[MeSH Terms] OR ("saint"[All Fields] AND "kitts"[All Fields] AND "nevis"[All Fields]) OR "saint kitts and nevis"[All Fields] OR ("st"[All Fields] AND "kitts"[All Fields] AND "nevis"[All Fields]) OR "st kitts and nevis"[All Fields])) OR ("saint kitts and nevis"[MeSH Terms] OR ("saint"[All Fields] AND "kitts"[All Fields] AND "nevis"[All Fields]) OR "saint kitts and nevis"[All Fields])) OR (("saints"[MeSH Terms] OR "saints"[All Fields] OR "saint"[All Fields]) AND Kitts[All Fields])) OR ("saint lucia"[MeSH Terms] OR ("saint"[All Fields] AND "lucia"[All Fields]) OR "saint lucia"[All Fields] OR ("st"[All Fields] AND "lucia"[All Fields]) OR "st lucia"[All Fields])) OR ("saint lucia"[MeSH Terms] OR ("saint"[All Fields] AND "lucia"[All Fields]) OR "saint lucia"[All Fields])) OR ("saint vincent and the grenadines"[MeSH Terms] OR ("saint"[All Fields] AND "vincent"[All Fields] AND "grenadines"[All Fields]) OR "saint vincent and the grenadines"[All Fields] OR ("st"[All Fields] AND "vincent"[All Fields] AND "grenadines"[All Fields]) OR "st vincent and the grenadines"[All Fields])) OR (St. Vincent[Author] OR St. Vincent[Investigator])) OR ("saint vincent and the grenadines"[MeSH Terms] OR ("saint"[All Fields] AND "vincent"[All Fields] AND "grenadines"[All Fields]) OR "saint vincent and the grenadines"[All Fields])) OR Saint Vincent[Author]) OR ("saint vincent and the grenadines"[MeSH Terms] OR ("saint"[All Fields] AND "vincent"[All Fields] AND "grenadines"[All Fields]) OR "saint vincent and the grenadines"[All Fields] OR "grenadines"[All Fields])) OR ("suriname"[MeSH Terms] OR "suriname"[All Fields])) OR ("thailand"[MeSH Terms] OR "thailand"[All Fields])) OR ("tunisia"[MeSH Terms] OR "tunisia"[All Fields])) OR ("turkey"[MeSH Terms] OR "turkey"[All Fields])) OR ("uruguay"[MeSH Terms] OR "uruguay"[All Fields])) OR ("venezuela"[MeSH Terms] OR "venezuela"[All Fields]))))

## CINAHL search strategy

# Query Limiters/Expanders

S44 S39 and S43 Search modes - Boolean/Phrase

Limiters - Published Date from: 20000101-20121231

S43 S40 or S41 or S42 Search modes - Boolean/Phrase

S42 Albania OR Algeria OR Samoa OR Antigua OR Barbuda OR Argentina OR Azerbaijan OR Belarus OR Bosnia OR Herzegovina OR Botswana OR Brazil OR Bulgaria OR Chile OR China OR Colombia OR Costa Rica OR Cuba OR Dominica OR Dominican Republic OR Ecuador OR Gabon OR Grenada OR Iran OR Jamaica OR Jordan OR Kazakhstan OR Latvia OR Lebanon OR Libya OR Lithuania OR Macedonia OR Malaysia OR Maldives OR Mauritius OR Mayotte OR Mexico OR Montenegro OR Namibia OR Palau OR Panama OR Peru OR Romania OR Russian Federation OR SerbiaOR Seychelles OR South Africa OR St. Kitts and Nevis OR St. Lucia OR St. Vincent OR Grenadines OR Suriname OR Thailand OR Tunisia OR Turkey OR Uruguay OR Venezuela Search modes - Boolean/Phrase

S41 Angola OR Armenia OR Belize OR Bhutan OR Bolivia OR Cameroon OR Cape Verde OR Congo, Rep OR Côte d'Ivoire OR Djibouti OR Egypt OR El Salvador OR Fiji OR Georgia OR Ghana OR Guatemala OR Guyana OR Honduras OR Indonesia OR India OR Iraq OR Kiribati OR Kosovo OR Lao PDR OR Lesotho OR Marshall Islands OR Mauritania OR Micronesia OR Moldova OR Mongolia OR Morocco OR Nicaragua OR Nigeria OR Pakistan OR Papua New Guinea OR Paraguay OR Philippines OR Samoa OR São Tomé and Principe OR Senegal OR Solomon Islands OR Sri Lanka OR Sudan OR Swaziland OR Syria* OR Timor-Leste OR Tonga OR Turkmenistan OR Tuvalu OR Ukraine OR Uzbekistan OR Vanuatu OR Vietnam OR Gaza OR Yemen OR Zambia Search modes - Boolean/Phrase

S40 Afghanistan OR Bangladesh OR Benin OR Burkina Faso OR Burundi OR Cambodia OR Central African Republic OR Chad OR Comoros OR Congo, Dem. Rep OR Eritrea OR Ethiopia OR Gambia, The OR Guinea OR Guinea-Bisau OR Haiti OR Kenya OR Korea, Dem Rep OR Kyrgyz Republic OR Liberia OR Madagascar OR Malawi OR Mali OR Mozambique OR Myanmar OR Nepal OR Niger OR Rwanda OR Sierra Leone OR Somalia OR Tajikistan OR Tanzania OR Togo OR Uganda OR Zimbabwe Search modes - Boolean/Phrase

S39 S8 or S13 or S19 or S25 or S38 Search modes - Boolean/Phrase

S38 S26 or S27 or S28 or S29 or S30 or S31 or S32 or S33 or S34 or S35 or S36 or S37 Search modes - Boolean/Phrase

S37 traditional birth attendant Search modes - Boolean/Phrase

S36 (attend* OR unattend*) N2 (birth* OR delivery or labo#r) Search modes - Boolean/Phrase

S35 unattended birth Search modes - Boolean/Phrase

S34 (MH "Lay Midwives") OR "birth attendant" Search modes - Boolean/Phrase

S33 (MH "Delivery Rooms") OR (MH "Alternative Birth Centers") Search modes - Boolean/Phrase

S32 (MH "Pregnancy, Ectopic") OR (MH "Pregnancy Complications, Cardiovascular+") OR (MH "Pregnancy Complications, Neoplastic+") OR (MH "Puerperal Disorders+") Search modes - Boolean/Phrase

S31 (MH "Intrapartum Care") OR (MH "Obstetric Care") OR (MH "Delivery") Search modes - Boolean/Phrase

S30 MM "Management of Labor" Search modes - Boolean/Phrase

S29 (pro#long* OR obstruct*) N2 (deliver* OR labo#r) Search modes - Boolean/Phrase

S28 "obstructed labor" Search modes - Boolean/Phrase

S27 Miscarriage Search modes - Boolean/Phrase

S26 (MH "Abortion, Spontaneous") Search modes - Boolean/Phrase

S25 S20 or S21 or S22 or S23 or S24 Search modes - Boolean/Phrase

S24 pre#eclampsia Search modes - Boolean/Phrase

S23 HELLP Search modes - Boolean/Phrase

S22 (MH "Eclampsia+") OR (MH "Pre-Eclampsia+") Search modes - Boolean/Phrase

S21 Eclampsia Search modes - Boolean/Phrase

S20 (MH "Pregnancy-Induced Hypertension") Search modes - Boolean/Phrase

S19 S14 or S15 or S17 or S18 Search modes - Boolean/Phrase

S18 "post#partum h#emorrhage" Search modes - Boolean/Phrase

S17 S2 N2 S16 Search modes - Boolean/Phrase

S16 h#emorrhage Search modes - Boolean/Phrase

S15 (MH "Postpartum Hemorrhage") Search modes - Boolean/Phrase

S14 postpartum hemorrhage Search modes - Boolean/Phrase

S13 S9 or S10 or S12 Search modes - Boolean/Phrase

S12 S2 N2 S11 Search modes - Boolean/Phrase

S11 an#emia Search modes - Boolean/Phrase

S10 MM "Pregnancy Complications, Hematologic" Search modes - Boolean/Phrase

S9 maternal anemia Search modes - Boolean/Phrase

S8 S1 or S4 or S5 or S6 or S7 Search modes - Boolean/Phrase

S7 MM "Pregnancy Complications, Infectious" Search modes - Boolean/Phrase

S6 infection in pregnancy Search modes - Boolean/Phrase

S5 "maternal infection" Search modes - Boolean/Phrase

S4 S2 N2 S3 Search modes - Boolean/Phrase

S3 (infect* OR sepsis OR septic OR tubercul* OR pneumonia OR meningitis OR HIV) Search modes - Boolean/Phrase

S2 (pregnan* OR maternal OR obstetric* OR puerper* OR partum OR birth OR childbirth) Search modes - Boolean/Phrase

S1 (MH "Chorioamnionitis") Search modes - Boolean/Phrase

## Embase search strategy

1 (Albania or Algeria or Samoa or Antigua or Barbuda or Argentina or Azerbaijan or Belarus or Bosnia or Herzegovina or Botswana or Brazil or Bulgaria or Chile or China or Colombia or Costa Rica or Cuba or Dominica or Dominican Republic or Ecuador or Gabon or Grenada or Iran or Jamaica or Jordan or Kazakhstan or Latvia or Lebanon or Libya or Lithuania or Macedonia or Malaysia or Maldives or Mauritius or Mayotte or Mexico or Montenegro or Namibia or Palau or Panama or Peru or Romania or Russian Federation or Russia or Serbia or Seychelles or South Africa or St Kitts or Nevis or St Lucia or St Vincent or Grenadines or Suriname or Thailand or Tunisia or Turkey or Uruguay or Venezuela).mp. [mp=title, abstract, subject headings, heading word, drug trade name, original title, device manufacturer, drug manufacturer, device trade name, keyword]

2 (Angola or Armenia or Belize or Bhutan or Bolivia or Cameroon or Cape Verde or Congo, or Cote d'Ivoire or Ivory Coast or Djibouti or Egypt or Arab Republic or El Salvador or Fiji or Georgia or Ghana or Guatemala or Guyana or Honduras or Indonesia or India or Iraq or Kiribati or Kosovo or Lao PDR or Lesotho or Marshall Islands or Mauritania or Micronesia or Moldova or Mongolia or Morocco or Nicaragua or Nigeria or Pakistan or Papua New Guinea or Paraguay or Philippines or Samoa or Sao Tome or Principe or Senegal or Solomon Islands or Sri Lanka or Sudan or Swaziland or Syrian Arab Republic or Timor-Leste or Tonga or Turkmenistan or Tuvalu or Ukraine or Uzbekistan or Vanuatu or Vietnam or West Bank or Gaza or Yemen or Zambia).mp. [mp=title, abstract, subject headings, heading word, drug trade name, original title, device manufacturer, drug manufacturer, device trade name, keyword]

3 (Afghanistan or Bangladesh or Benin or Burkina Faso or Burundi or Cambodia or Central African Republic or Chad or Comoros or Congo or Eritrea or Ethiopia or Gambia or Guinea or Bisau or Haiti or Kenya or Korea or Kyrgyz or Liberia or Madagascar or Malawi or Mali or Mozambique or Myanmar or Nepal or Niger or Rwanda or Sierra Leone or Somalia or Tajikistan or Tanzania or Togo or Uganda or Zimbabwe).mp. [mp=title, abstract, subject headings, heading word, drug trade name, original title, device manufacturer, drug manufacturer, device trade name, keyword]

4 (Africa or sahara* or low income country or low income countries or middle income country or middle income countries or south america or central america or latin america or carribean).mp. [mp=title, abstract, subject headings, heading word, drug trade name, original title, device manufacturer, drug manufacturer, device trade name, keyword]

5 exp Developing Countries/

6 (#1 or #2 or #3 or #4 or #5).mp. [mp=title, abstract, subject headings, heading word, drug trade name, original title, device manufacturer, drug manufacturer, device trade name, keyword]

7 limit 6 to (human and yr="2000 -Current")

8 maternal infection.mp.

9 chorioamnionitis.mp.

10 exp maternal disease/ or exp intrauterine infection/

11 (pregnan* or maternal or obstetric* or puerper* or partum or birth or childbirth or prenatal or postnatal or natal).mp. [mp=title, abstract, subject headings, heading word, drug trade name, original title, device manufacturer, drug manufacturer, device trade name, keyword]

12 (infect* or sepsis or septic or tubercul* or pneumonia or meningitis or HIV).mp. [mp=title, abstract, subject headings, heading word, drug trade name, original title, device manufacturer, drug manufacturer, device trade name, keyword]

13 (#11 adj3 #12).mp. [mp=title, abstract, subject headings, heading word, drug trade name, original title, device manufacturer, drug manufacturer, device trade name, keyword]

14 maternal anemia.mp.

15 exp PREGNANCY COMPLICATIONS, HEMATOLOGIC/

16 (anemi* or anaemi* or hemoglobin or haemoglobin).mp. [mp=title, abstract, subject headings, heading word, drug trade name, original title, device manufacturer, drug manufacturer, device trade name, keyword]

17 (#11 adj3 #16).mp. [mp=title, abstract, subject headings, heading word, drug trade name, original title, device manufacturer, drug manufacturer, device trade name, keyword]

18 exp postpartum hemorrhage/

19 ((maternal or obstetric* or puerper* or partum or birth or childbirth or postnatal) adj2 (bleed or bleeding or hemorrhage or haemorrhage)).mp. [mp=title, abstract, subject headings, heading word, drug trade name, original title, device manufacturer, drug manufacturer, device trade name, keyword]

20 exp "eclampsia and preeclampsia"/ or exp eclampsia/

21 (eclampsia or pre-ecalmapsia or preeclampsia or HELLP).ti,ab.

22 miscarriage.ti,ab.

23 exp SPONTANEOUS ABORTION/

24 obstructed labor.mp.

25 exp LABOR OBSTRUCTION/

26 ((obstruct* or prolong*) adj2 (labour or labor or delivery or birth or childbirth)).ti,ab.

27 exp LABOR MANAGEMENT/

28 exp intrapartum care/

29 exp perinatal care/

30 exp DELIVERY ROOM/

31 exp HOME DELIVERY/

32 exp birthplace/

33 birth attendant*.mp.

34 place* of birth*.mp.

35 ((attend* or unattend* or alone or support) adj2 (Birth* or childbirth* or deliver*)).ti,ab.

36 *MATERNAL CARE/

37 8 or 9 or 10 or 13 or 14 or 15 or 17 or 18 or 19 or 20 or 21 or 22 or 23 or 24 or 25 or 26 or 27 or 28 or 29 or 30 or 31 or 32 or 33 or 34 or 35 or 36

38 clincal trial.mp.

39 phase 1 clinical trial/

40 phase 2 clinical trial/

41 controlled clinical trial/ or clinical trial/ or "controlled clinical trial (topic)"/

42 phase 3 clinical trial/

43 phase 4 clinical trial/

44 38 or 39 or 40 or 41 or 42 or 43

45 7 and 37

46 limit 45 to ((evidence based medicine or meta analysis or outcomes research or "systematic review") and yr="2000 -Current")

## PsycINFO search strategy

1 (Albania or Algeria or Samoa or Antigua or Barbuda or Argentina or Azerbaijan or Belarus or Bosnia or Herzegovina or Botswana or Brazil or Bulgaria or Chile or China or Colombia or Costa Rica or Cuba or Dominica or Dominican Republic or Ecuador or Gabon or Grenada or Iran or Jamaica or Jordan or Kazakhstan or Latvia or Lebanon or Libya or Lithuania or Macedonia or Malaysia or Maldives or Mauritius or Mayotte or Mexico or Montenegro or Namibia or Palau or Panama or Peru or Romania or Russian Federation or Russia or Serbia or Seychelles or South Africa or St Kitts or Nevis or St Lucia or St Vincent or Grenadines or Suriname or Thailand or Tunisia or Turkey or Uruguay or Venezuela).mp. [mp=title, abstract, heading word, table of contents, key concepts, original title, tests & measures] (51248) 2 (Angola or Armenia or Belize or Bhutan or Bolivia or Cameroon or Cape Verde or Congo, or Cote d'Ivoire or Ivory Coast or Djibouti or Egypt or Arab Republic or El Salvador or Fiji or Georgia or Ghana or Guatemala or Guyana or Honduras or Indonesia or India or Iraq or Kiribati or Kosovo or Lao PDR or Lesotho or Marshall Islands or Mauritania or Micronesia or Moldova or Mongolia or Morocco or Nicaragua or Nigeria or Pakistan or Papua New Guinea or Paraguay or Philippines or Samoa or Sao Tome or Principe or Senegal or Solomon Islands or Sri Lanka or Sudan or Swaziland or Syrian Arab Republic or Timor-Leste or Tonga or Turkmenistan or Tuvalu or Ukraine or Uzbekistan or Vanuatu or Vietnam or West Bank or Gaza or Yemen or Zambia).mp. [mp=title, abstract, heading word, table of contents, key concepts, original title, tests & measures] (28159) 3 (Afghanistan or Bangladesh or Benin or Burkina Faso or Burundi or Cambodia or Central African Republic or Chad or Comoros or Congo or Eritrea or Ethiopia or Gambia or Guinea or Bisau or Haiti or Kenya or Korea or Kyrgyz or Liberia or Madagascar or Malawi or Mali or Mozambique or Myanmar or Nepal or Niger or Rwanda or Sierra Leone or Somalia or Tajikistan or Tanzania or Togo or Uganda or Zimbabwe).mp. [mp=title, abstract, heading word, table of contents, key concepts, original title, tests & measures] (15936) 4 (Africa or sahara* or low income country or low income countries or middle income country or middle income countries or south america or central america or latin america or carribean).mp. [mp=title, abstract, heading word, table of contents, key concepts, original title, tests & measures] (13920) 5 exp Developing Countries/ (3010) 6 limit 2 to (human and yr="2000 - 2012") (18992) 7 limit 3 to (human and yr="2000 - 2012") (10958) 8 limit 4 to (human and yr="2000 - 2012") (10674) 9 limit 5 to (human and yr="2000 - 2012") (2363) 10 (#1 or #2 or #3 or #4 or #5).mp. [mp=title, abstract, heading word, table of contents, key concepts, original title, tests & measures] (906414) 11 limit 10 to (human and yr="2000 -Current") (510379) 12 maternal infection.mp. (118) 13 chorioamnionitis.mp. (40) 14 exp midwifery/ or exp obstetrical complications/ (1531) 15 miscarriage.mp. or exp Spontaneous Abortion/ (768) 16 (pregnan* or maternal or obstetric* or puerper* or partum or birth or childbirth or prenatal or postnatal or natal or post-partum).mp. [mp=title, abstract, heading word, table of contents, key concepts, original title, tests & measures] (84865) 17 (infect* or sepsis or septic or tubercul* or pneumonia or meningitis or HIV or hemorrhage or haemorrhage or bleed*).mp. [mp=title, abstract, heading word, table of contents, key concepts, original title, tests & measures] (49913) 18 (#18 adj3 #19).mp. [mp=title, abstract, heading word, table of contents, key concepts, original title, tests & measures] (2516) 19 (anemia or anaemia).mp. [mp=title, abstract, heading word, table of contents, key concepts, original title, tests & measures] (1011) 20 (#18 adj3 #21).mp. [mp=title, abstract, heading word, table of contents, key concepts, original title, tests & measures] (1230) 21 ((obstruc* or prolong*) adj3 (labour or labour or birth or delivery)).mp. [mp=title, abstract, heading word, table of contents, key concepts, original title, tests & measures] (43) 22 birth attendant*.mp. [mp=title, abstract, heading word, table of contents, key concepts, original title, tests & measures] (110) 23 childbirth.mp. [mp=title, abstract, heading word, table of contents, key concepts, original title, tests & measures] (2867) 24 *Birth/ (2853) 25 12 or 13 or 14 or 15 or 18 or 20 or 21 or 22 or 23 or 24 (10515) 26 11 and 25 (4257) 27 limit 26 to (human and ("reviews (maximizes sensitivity)" or "therapy (maximizes sensitivity)" or "qualitative (maximizes sensitivity)") and human and yr="2000 -Current") (3171) 28 limit 27 to (120 neonatal <birth to age 1 mo> or 200 adolescence <age 13 to 17 yrs> or 320 young adulthood <age 18 to 29 yrs> or 340 thirties <age 30 to 39 yrs> or 360 middle age <age 40 to 64 yrs>) (1139) 29 limit 28 to (100 childhood <birth to age 12 yrs> or 200 adolescence <age 13 to 17 yrs> or 320 young adulthood <age 18 to 29 yrs> or 340 thirties <age 30 to 39 yrs> or 360 middle age <age 40 to 64 yrs>) (1139) 30 limit 29 to yr="2000 - 2005" (339) 31 limit 29 to yr="2006 -Current" (800)

## Web of Knowledge search strategy

Set Results Search terms

# 69 8,903

#59 AND #6

Refined by: Web of Science Categories=( PUBLIC ENVIRONMENTAL OCCUPATIONAL HEALTH OR INFECTIOUS DISEASES OR OBSTETRICS GYNECOLOGY OR MEDICINE GENERAL INTERNAL OR TROPICAL MEDICINE OR SOCIAL SCIENCES BIOMEDICAL OR HEALTH POLICY SERVICES OR NURSING OR MEDICINE RESEARCH EXPERIMENTAL )

Databases=SCI-EXPANDED, SSCI, A&HCI Timespan=2000-01-01 - 2012-09-21

Lemmatization=On

# 68 1,248

#59 AND #6

Refined by: Web of Science Categories=( MEDICINE GENERAL INTERNAL )

Databases=SCI-EXPANDED, SSCI, A&HCI Timespan=2000-01-01 - 2012-09-21

Lemmatization=On

# 67 3,064

#59 AND #6

Refined by: Web of Science Categories=( PUBLIC ENVIRONMENTAL OCCUPATIONAL HEALTH )

Databases=SCI-EXPANDED, SSCI, A&HCI Timespan=2000-01-01 - 2012-09-21

Lemmatization=On

# 66 1,560

#59 AND #6

Refined by: Web of Science Categories=( IMMUNOLOGY )

Databases=SCI-EXPANDED, SSCI, A&HCI Timespan=2000-01-01 - 2012-09-21

Lemmatization=On

# 65 2,080

#59 AND #6

Refined by: Web of Science Categories=( INFECTIOUS DISEASES )

Databases=SCI-EXPANDED, SSCI, A&HCI Timespan=2000-01-01 - 2012-09-21

Lemmatization=On

# 64 10,555

#59 AND #6

Refined by: Web of Science Categories=( PUBLIC ENVIRONMENTAL OCCUPATIONAL HEALTH OR INFECTIOUS DISEASES OR OBSTETRICS GYNECOLOGY OR IMMUNOLOGY OR MEDICINE GENERAL INTERNAL OR TROPICAL MEDICINE OR PEDIATRICS OR VIROLOGY OR MICROBIOLOGY OR SOCIAL SCIENCES BIOMEDICAL OR PARASITOLOGY OR HEALTH POLICY SERVICES OR NUTRITION DIETETICS OR NURSING OR MEDICINE RESEARCH EXPERIMENTAL OR HEALTH CARE SCIENCES SERVICES OR REPRODUCTIVE BIOLOGY )

Databases=SCI-EXPANDED, SSCI, A&HCI Timespan=2000-01-01 - 2012-09-21

Lemmatization=On

# 63 10,852

#59 AND #6

Refined by: Web of Science Categories=( PUBLIC ENVIRONMENTAL OCCUPATIONAL HEALTH OR INFECTIOUS DISEASES OR OBSTETRICS GYNECOLOGY OR IMMUNOLOGY OR WOMEN S STUDIES OR MEDICINE GENERAL INTERNAL OR TROPICAL MEDICINE OR PEDIATRICS OR VIROLOGY OR MICROBIOLOGY OR SOCIAL SCIENCES BIOMEDICAL OR PARASITOLOGY OR HEALTH POLICY SERVICES OR NUTRITION DIETETICS OR NURSING OR MEDICINE RESEARCH EXPERIMENTAL OR BIOLOGY OR RESPIRATORY SYSTEM OR HEALTH CARE SCIENCES SERVICES OR DEMOGRAPHY OR ENVIRONMENTAL SCIENCES OR PSYCHOLOGY MULTIDISCIPLINARY OR REPRODUCTIVE BIOLOGY OR SURGERY ) AND Research Areas=( PUBLIC ENVIRONMENTAL OCCUPATIONAL HEALTH OR INFECTIOUS DISEASES OR OBSTETRICS GYNECOLOGY OR IMMUNOLOGY OR GENERAL INTERNAL MEDICINE OR TROPICAL MEDICINE OR PEDIATRICS OR VIROLOGY OR MICROBIOLOGY OR BIOMEDICAL SOCIAL SCIENCES OR HEALTH CARE SCIENCES SERVICES OR PARASITOLOGY OR NUTRITION DIETETICS OR NURSING OR RESEARCH EXPERIMENTAL MEDICINE OR LIFE SCIENCES BIOMEDICINE OTHER TOPICS OR REPRODUCTIVE BIOLOGY OR WOMEN S STUDIES )

Databases=SCI-EXPANDED, SSCI, A&HCI Timespan=2000-01-01 - 2012-09-21

Lemmatization=On

# 62 11,111

#59 AND #6

Refined by: Web of Science Categories=( PUBLIC ENVIRONMENTAL OCCUPATIONAL HEALTH OR INFECTIOUS DISEASES OR OBSTETRICS GYNECOLOGY OR IMMUNOLOGY OR WOMEN S STUDIES OR MEDICINE GENERAL INTERNAL OR TROPICAL MEDICINE OR PEDIATRICS OR VIROLOGY OR MICROBIOLOGY OR SOCIAL SCIENCES BIOMEDICAL OR PARASITOLOGY OR HEALTH POLICY SERVICES OR NUTRITION DIETETICS OR NURSING OR MEDICINE RESEARCH EXPERIMENTAL OR BIOLOGY OR RESPIRATORY SYSTEM OR HEALTH CARE SCIENCES SERVICES OR DEMOGRAPHY OR ENVIRONMENTAL SCIENCES OR PSYCHOLOGY MULTIDISCIPLINARY OR REPRODUCTIVE BIOLOGY OR SURGERY )

Databases=SCI-EXPANDED, SSCI, A&HCI Timespan=2000-01-01 - 2012-09-21

Lemmatization=On

# 61 5,217

#59 AND #6

Refined by: Web of Science Categories=( OBSTETRICS GYNECOLOGY OR WOMEN S STUDIES OR MEDICINE GENERAL INTERNAL OR HEMATOLOGY OR TROPICAL MEDICINE OR PEDIATRICS OR SOCIAL SCIENCES BIOMEDICAL OR SOCIAL SCIENCES INTERDISCIPLINARY OR SOCIOLOGY OR SOCIAL ISSUES )

Databases=SCI-EXPANDED, SSCI, A&HCI Timespan=2000-01-01 - 2012-09-21

Lemmatization=On

# 60 13,054

#59 AND #6

Databases=SCI-EXPANDED, SSCI, A&HCI Timespan=2000-01-01 - 2012-09-21

Lemmatization=On

# 59 95,097

#58 OR #33 OR #29 OR #22 OR #18

Databases=SCI-EXPANDED, SSCI, A&HCI Timespan=2000-01-01 - 2012-09-21

Lemmatization=On

# 58 37,609

#57 OR #56 OR #55 OR #54 OR #53 OR #52 OR #51 OR #47 OR #46 OR #45 OR #44 OR #43 OR #42 OR #41 OR #40 OR #39 OR #38 OR #37 OR #36 OR #35 OR #34

Databases=SCI-EXPANDED, SSCI, A&HCI Timespan=2000-01-01 - 2012-09-21

Lemmatization=On

# 57 395

Topic=((*attend* childbirth*))

Databases=SCI-EXPANDED, SSCI, A&HCI Timespan=2000-01-01 - 2012-09-21

Lemmatization=On

# 56 2,833

Topic=((*attend* birth))

Databases=SCI-EXPANDED, SSCI, A&HCI Timespan=2000-01-01 - 2012-09-21

Lemmatization=On

# 55 1,592

Topic=("maternal care")

Databases=SCI-EXPANDED, SSCI, A&HCI Timespan=2000-01-01 - 2012-09-21

Lemmatization=On

# 54 3,944

Topic=(midwife)

Databases=SCI-EXPANDED, SSCI, A&HCI Timespan=2000-01-01 - 2012-09-21

Lemmatization=On

# 53 529

Topic=("birth attendant*")

Databases=SCI-EXPANDED, SSCI, A&HCI Timespan=2000-01-01 - 2012-09-21

Lemmatization=On

# 52 587

Topic=("place of birth")

Databases=SCI-EXPANDED, SSCI, A&HCI Timespan=2000-01-01 - 2012-09-21

Lemmatization=On

# 51 2,632

#13 AND #50

Databases=SCI-EXPANDED, SSCI, A&HCI Timespan=2000-01-01 - 2012-09-21

Lemmatization=On

# 50 8,104

#49 OR #48

Databases=SCI-EXPANDED, SSCI, A&HCI Timespan=2000-01-01 - 2012-09-21

Lemmatization=On

# 49 8,104

Topic=((labour management))

Databases=SCI-EXPANDED, SSCI, A&HCI Timespan=2000-01-01 - 2012-09-21

Lemmatization=On

# 48 8,104

Topic=((labor management))

Databases=SCI-EXPANDED, SSCI, A&HCI Timespan=2000-01-01 - 2012-09-21

Lemmatization=On

# 47 1,002

Topic=("obstetric care")

Databases=SCI-EXPANDED, SSCI, A&HCI Timespan=2000-01-01 - 2012-09-21

Lemmatization=On

# 46 195

Topic=("intrapartum care")

Databases=SCI-EXPANDED, SSCI, A&HCI Timespan=2000-01-01 - 2012-09-21

Lemmatization=On

# 45 17,221

Topic=(pregnan* complicat*)

Databases=SCI-EXPANDED, SSCI, A&HCI Timespan=2000-01-01 - 2012-09-21

Lemmatization=On

# 44 7

Topic=("complication* of labour")

Databases=SCI-EXPANDED, SSCI, A&HCI Timespan=2000-01-01 - 2012-09-21

Lemmatization=On

# 43 32

Topic=("complication* of labor")

Databases=SCI-EXPANDED, SSCI, A&HCI Timespan=2000-01-01 - 2012-09-21

Lemmatization=On

# 42 2,810

Topic=(complication* of labor)

Databases=SCI-EXPANDED, SSCI, A&HCI Timespan=2000-01-01 - 2012-09-21

Lemmatization=On

# 41 1,755

Topic=(dystocia)

Databases=SCI-EXPANDED, SSCI, A&HCI Timespan=2000-01-01 - 2012-09-21

Lemmatization=On

# 40 465

Topic=(OBSTRUCT* LABOUR)

Databases=SCI-EXPANDED, SSCI, A&HCI Timespan=2000-01-01 - 2012-09-21

Lemmatization=On

# 39 1,223

Topic=(PROLONG* LABOUR)

Databases=SCI-EXPANDED, SSCI, A&HCI Timespan=2000-01-01 - 2012-09-21

Lemmatization=On

# 38 1,223

Topic=(PROLONG* LABOR)

Databases=SCI-EXPANDED, SSCI, A&HCI Timespan=2000-01-01 - 2012-09-21

Lemmatization=On

# 37 465

Topic=(OBSTRUCT* LABOR)

Databases=SCI-EXPANDED, SSCI, A&HCI Timespan=2000-01-01 - 2012-09-21

Lemmatization=On

# 36 1,476

Topic=("SPONTANEOUS ABORTIONS")

Databases=SCI-EXPANDED, SSCI, A&HCI Timespan=2000-01-01 - 2012-09-21

Lemmatization=On

# 35 3,363

Topic=("SPONTANEOUS ABORTION")

Databases=SCI-EXPANDED, SSCI, A&HCI Timespan=2000-01-01 - 2012-09-21

Lemmatization=On

# 34 4,380

Topic=(miscarriage*) AND Topic=(pregnan*)

Databases=SCI-EXPANDED, SSCI, A&HCI Timespan=2000-01-01 - 2012-09-21

Lemmatization=On

# 33 19,698

#32 OR #31 OR #30

Databases=SCI-EXPANDED, SSCI, A&HCI Timespan=2000-01-01 - 2012-09-21

Lemmatization=On

# 32 8,922

Topic=(hypertens*) AND Topic=(pregnan*)

Databases=SCI-EXPANDED, SSCI, A&HCI Timespan=2000-01-01 - 2012-09-21

Lemmatization=On

# 31 1,158

Topic=(HELLP)

Databases=SCI-EXPANDED, SSCI, A&HCI Timespan=2000-01-01 - 2012-09-21

Lemmatization=On

# 30 15,082

Topic=(*eclampsia*)

Databases=SCI-EXPANDED, SSCI, A&HCI Timespan=2000-01-01 - 2012-09-21

Lemmatization=On

# 29 2,959

#28 OR #27 OR #26 OR #25 OR #24 OR #23

Databases=SCI-EXPANDED, SSCI, A&HCI Timespan=2000-01-01 - 2012-09-21

Lemmatization=On

# 28 802

Topic=(*natal* h?emorrhage)

Databases=SCI-EXPANDED, SSCI, A&HCI Timespan=2000-01-01 - 2012-09-21

Lemmatization=On

# 27 479

Topic=(obstetric h?emorrhage)

Databases=SCI-EXPANDED, SSCI, A&HCI Timespan=2000-01-01 - 2012-09-21

Lemmatization=On

# 26 0 Topic=(obstetric h?emorhage)

Databases=SCI-EXPANDED, SSCI, A&HCI Timespan=2000-01-01 - 2012-09-21

Lemmatization=On

# 25 697

Topic=(postpartum bleed*)

Databases=SCI-EXPANDED, SSCI, A&HCI Timespan=2000-01-01 - 2012-09-21

Lemmatization=On

# 24 510

Topic=("postpartum haemorrhage".)

Databases=SCI-EXPANDED, SSCI, A&HCI Timespan=2000-01-01 - 2012-09-21

Lemmatization=On

# 23 1,353

Topic=("postpartum hemorrhage".)

Databases=SCI-EXPANDED, SSCI, A&HCI Timespan=2000-01-01 - 2012-09-21

Lemmatization=On

# 22 1,835

#21 OR #19

Databases=SCI-EXPANDED, SSCI, A&HCI Timespan=2000-01-01 - 2012-09-21

Lemmatization=On

# 21 1,835

#13 AND #20

Databases=SCI-EXPANDED, SSCI, A&HCI Timespan=2000-01-01 - 2012-09-21

Lemmatization=On

# 20 18,960

Topic=(an?emi* OR h?emoglobin)

Databases=SCI-EXPANDED, SSCI, A&HCI Timespan=2000-01-01 - 2012-09-21

Lemmatization=On

# 19 113

Topic=("maternal an?emia")

Databases=SCI-EXPANDED, SSCI, A&HCI Timespan=2000-01-01 - 2012-09-21

Lemmatization=On

# 18 45,324

#17 OR #16 OR #15 OR #12 OR #10 OR #9 OR #8 OR #7

Databases=SCI-EXPANDED, SSCI, A&HCI Timespan=2000-01-01 - 2012-09-21

Lemmatization=On

# 17 43

Topic=("PUERPERAL INFECTION")

Databases=SCI-EXPANDED, SSCI, A&HCI Timespan=2000-01-01 - 2012-09-21

Lemmatization=On

# 16 246

Topic=("infection in pregnancy")

Databases=SCI-EXPANDED, SSCI, A&HCI Timespan=2000-01-01 - 2012-09-21

Lemmatization=On

# 15 43,468

#14 AND #13

Databases=SCI-EXPANDED, SSCI, A&HCI Timespan=2000-01-01 - 2012-09-21

Lemmatization=On

# 14 819,451

Topic=(infect* OR sepsis OR septic OR tubercul* OR pneumonia OR meningitis OR HIV)

Databases=SCI-EXPANDED, SSCI, A&HCI Timespan=2000-01-01 - 2012-09-21

Lemmatization=On

# 13 417,789

Topic=(pregnan* OR maternal OR obstetric* OR puerper* OR partum OR birth OR childbirth OR prenatal OR postnatal OR *natal*)

Databases=SCI-EXPANDED, SSCI, A&HCI Timespan=2000-01-01 - 2012-09-21

Lemmatization=On

# 12 925

Topic=("INTRAUTERINE INFECTION")

Databases=SCI-EXPANDED, SSCI, A&HCI Timespan=2000-01-01 - 2012-09-21

Lemmatization=On

# 11 2,514

Topic=(INTRAUTERINE INFECTION)

Databases=SCI-EXPANDED, SSCI, A&HCI Timespan=2000-01-01 - 2012-09-21

Lemmatization=On

# 10 1,013

Topic=(FEMALE GENITAL TRACT INFECTION)

Databases=SCI-EXPANDED, SSCI, A&HCI Timespan=2000-01-01 - 2012-09-21

Lemmatization=On

# 9 155

Topic=(FEMALE GENITAL TRACT INFLAMMATION)

Databases=SCI-EXPANDED, SSCI, A&HCI Timespan=2000-01-01 - 2012-09-21

Lemmatization=On

# 8 1,821

Topic=(chorioamnionitis)

Databases=SCI-EXPANDED, SSCI, A&HCI Timespan=2000-01-01 - 2012-09-21

Lemmatization=On

# 7 7,405

Topic=(maternal infection*)

Databases=SCI-EXPANDED, SSCI, A&HCI Timespan=2000-01-01 - 2012-09-21

Lemmatization=On

# 6 848,085

#5

Databases=SCI-EXPANDED, SSCI, A&HCI Timespan=2000-01-01 - 2012-09-21

Lemmatization=On

# 5 1,375,550

#4 OR #3 OR #2 OR #1

Databases=SCI-EXPANDED, SSCI, A&HCI Timespan=All Years

Lemmatization=On

# 4 217,559

Topic=(Africa OR *sahara* OR "low income country" OR "low income countries" OR "middle income country" OR "middle income countries" OR "south america" OR "central america" OR "latin america" OR carribean)

Databases=SCI-EXPANDED, SSCI, A&HCI Timespan=All Years

Lemmatization=On

# 3 264,996

Topic=(Afghanistan OR Bangladesh OR Benin OR Burkina Faso OR Burundi OR Cambodia OR Central African Republic OR Chad OR Comoros OR Congo, Dem. Rep OR Eritrea OR Ethiopia OR Gambia, The OR Guinea OR Guinea-Bisau OR Haiti OR Kenya OR Korea, Dem Rep OR Kyrgyz Republic OR Liberia OR Madagascar OR Malawi OR Mali OR Mozambique OR Myanmar OR Nepal OR Niger OR Rwanda OR Sierra Leone OR Somalia OR Tajikistan OR Tanzania OR Togo OR Uganda OR Zimbabwe)

Databases=SCI-EXPANDED, SSCI, A&HCI Timespan=All Years

Lemmatization=On

# 2 380,742

Topic=(Angola OR Armenia OR Belize OR Bhutan OR Bolivia OR Cameroon OR Cape Verde OR Congo, Rep OR C??te d'Ivoire OR Djibouti OR Egypt OR El Salvador OR Fiji OR Georgia OR Ghana OR Guatemala OR Guyana OR Honduras OR Indonesia OR India OR Iraq OR Kiribati OR Kosovo OR Lao PDR OR Lesotho OR Marshall Islands OR Mauritania OR Micronesia OR Moldova OR Mongolia OR Morocco OR Nicaragua OR Nigeria OR Pakistan OR Papua New Guinea OR Paraguay OR Philippines OR Samoa OR S??o Tom?? and Principe OR Senegal OR Solomon Islands OR Sri Lanka OR Sudan OR Swaziland OR Syria* OR Timor-Leste OR Tonga OR Turkmenistan OR Tuvalu OR Ukraine OR Uzbekistan OR Vanuatu OR Vietnam OR Gaza OR Yemen OR Zambia)

Databases=SCI-EXPANDED, SSCI, A&HCI Timespan=All Years

Lemmatization=On

# 1 732,634

Topic=(Albania OR Algeria OR Samoa OR Antigua OR Barbuda OR Argentina OR Azerbaijan OR Belarus OR Bosnia OR Herzegovina OR Botswana OR Brazil OR Bulgaria OR Chile OR China OR Colombia OR Costa Rica OR Cuba OR Dominica OR Dominican Republic OR Ecuador OR Gabon OR Grenada OR Iran OR Jamaica OR Jordan OR Kazakhstan OR Latvia OR Lebanon OR Libya OR Lithuania OR Macedonia OR Malaysia OR Maldives OR Mauritius OR Mayotte OR Mexico OR Montenegro OR Namibia OR Palau OR Panama OR Peru OR Romania OR Russian Federation OR SerbiaOR Seychelles OR South Africa OR St. Kitts and Nevis OR St. Lucia OR St. Vincent OR Grenadines OR Suriname OR Thailand OR Tunisia OR Turkey OR Uruguay OR Venezuela)

Databases=SCI-EXPANDED, SSCI, A&HCI Timespan=All Years

Lemmatization=On

.

## Popline search strategy

This database does not allow complicated searching. Searches are limited to 1 line with limited Boolean options. The subject search option was therefore used as this has a range of maternal and child health options . Phrases and subjects are listed below

1 Subject pregnancy and childbirth complications

2. Safe motherhood

3. “postpartum hemorrhage”

4. Antenatal care –

5. Post-partum care – post partum women

6. Maternal care (limits -– developing countries – health services)

7. Maternal care (limits– developing countries – delivery of health services)

8. Maternal care (limits treatment – developing countries )

9. Maternal care (limits evaluation – developing countries )

10. Maternal mortality (limits developing countries)

11. Post-partum care

12. Contraception for post-partum women

13. Early detection (limits developing countries)

14. Danger signs (limits developing countries and socioeconomic factors)

## LILACS

A combination of search terms were used all of which were limited to items about pregnancy. Search terms were: anemia or anaemia, birth attendant, hemorrhage or haemorrhage, infections terms, intrauterine infection, intrapartum care, maternal infection, maternal mortality, miscarriage, pre-eclampsia.
